# Local agro-pastoralists’ perspectives on forage species diversity, habitat distributions, abundance trends and ecological drivers for sustainable livestock production in West Africa

**DOI:** 10.1038/s41598-019-38636-1

**Published:** 2019-02-08

**Authors:** John-Baptist S. N. Naah, Boris Braun

**Affiliations:** 1 Institute of Geography, University of Cologne, Albertus Magnus Platz, D-50923 Cologne, Germany; 2 Center for Development Research (ZEF), University of Bonn, Genscherallee 3, D-53113 Bonn, Germany

## Abstract

Despite the importance of local ecological knowledge of forage plants, there has been little discussion on how local agro-pastoralists perceive forage species diversity, abundance trends, habitat distributions and ecological drivers influencing changing abundance trends over time in rural West Africa’s savannas. In estimating, assessing and investigating the ecological variables, we performed elaborate ethnobotanical surveys in seven villages in northern Ghana and nine villages in southern-central Burkina Faso. Data were analyzed using descriptive statistics, bivariate correlation analysis and cognitive salience index calculations to disentangle the dynamics of local responses to ecological variables considered in this study. Our results revealed that agro-pastoralists exhibited extensive knowledge on forage species diversity, habitat types, abundance trends and ecological drivers. According to agro-pastoralists interviewed, about 82 percent of all forage species known to them were commonly available in local landscapes, while a majority of our interviewees indicated that available forage resources have shown a gradually increasing trend over the past few years. Rainfall variability, tree cutting and drought were the topmost perceived threats causing changes in the trends of forage species abundance. Given our findings, local perceptions of agro-pastoralists could have substantial practical implications in favor of forage-related biodiversity conservation and sustainable livestock production.

## Introduction

A plethora of literature abounds with evidence that future projections and scenarios of global climatic conditions point to increasing incidences of unpredictable precipitation patterns, drought spells, overgrazing, severe mean annual temperature rises, intermittent floods and land degradation particularly in global drylands^1–4^, and such changes are unbeneficial^4^. Hence, tropical West Africa, in particular, has been experiencing a significant increase in such challenging anthropogenic and natural incidences^5^ and human population growth^6^. This results in severe changes in the vegetation composition and species cover in West African savanna ecosystems^7^. Smallholder farmers in this region and other developing countries are thus considered as disproportionately vulnerable to climate change owing to its direct effect on their crop and animal productivity, as well as negative consequences on their food security, income and general well-being^8^. Being able to make use of a diverse portfolio of management strategies, and to have diversified sources of income, may therefore greatly increase (agro)pastoralists’ livelihood security, particularly in a highly variable environment^9,10^. Climate change and variability, especially scarcity of water, might lead to reduced supply of essential ecosystem services including forage provision for livestock production while increasing demand for these ecosystem services from the vegetation.

Apart from climate change and variability, smallholder farmers are also faced with non-climatic stressors^11^. For instance, overgrazing is a commonly observed land use challenge in communal rangelands caused by domestic livestock reared among smallholder farmers, as it was reported by Bahru *et al*.^12^ in semi-arid localities in Ethiopia. However, many local smallholder farmers in West African savannas largely engage in rain-fed agricultural practices as their main source of employment, food and income^13^. While dryland rangelands do support approximately 50 percent of global livestock production^14^, continued forage availability for livestock production is highly crucial since about 45 percent of rural households heavily rely upon sources of livestock-related income in typical West African settings^15^. The delivery of these forage services from dryland rangelands is mainly dependent on rangelands’ floristic composition^16,17^.

To ensure effective sustainability and biodiversity conservation efforts in natural resources management, understanding social-ecological systems (SES) is vitally critical^18^. Studies have shown that land users’ local ecological knowledge (LEK) on forage plants is of critical importance for their adaptive rangeland management^19,20^. Research has also shown that agro-pastoralists’ LEK of forage resources is an essential component of management decisions^20,21^, and plays a crucial role in agro-pastoralists’ adaptation to climate change^22^. Identifying crucial aspects of an adaptive natural resource management appears a promising approach for developing mitigation strategies for negative consequences of projected climate change in West Africa.

Despite the importance of LEK on forage plants, very little research has been done on how local agro-pastoralists perceive forage species diversity, abundance trends, habitat distributions and ecological drivers influencing their changing numbers over time in rural West Africa’s savannas. As local knowledge tends to be unevenly distributed among local resource users and better understood in a particular context^23^, we expect local agro-pastoralists’ perspectives on the above-referenced ecological variables to be different. This ethnobotanical study is not intended to investigate causalities of such ecological variables with respect to plant citations provided by local agro-pastoralists in the research region. The main research question we address in this paper is: ‘what are the important ecological variables from the perspectives of local ago-pastoralists for sustainable livestock production in West Africa?’ We set the following specific research objectives, which include:

- Estimation of forage species diversity based on local agro-pastoralists’ LEK.
- Assessment of abundance and trends of forage resources based on perceptions of local agro-pastoralists.
- Investigation of perceived local ecological drivers responsible for changes in abundance and trends of forage plants and local conservation measures.

## Materials and Methods

### Description of study areas

**Figure 1:**
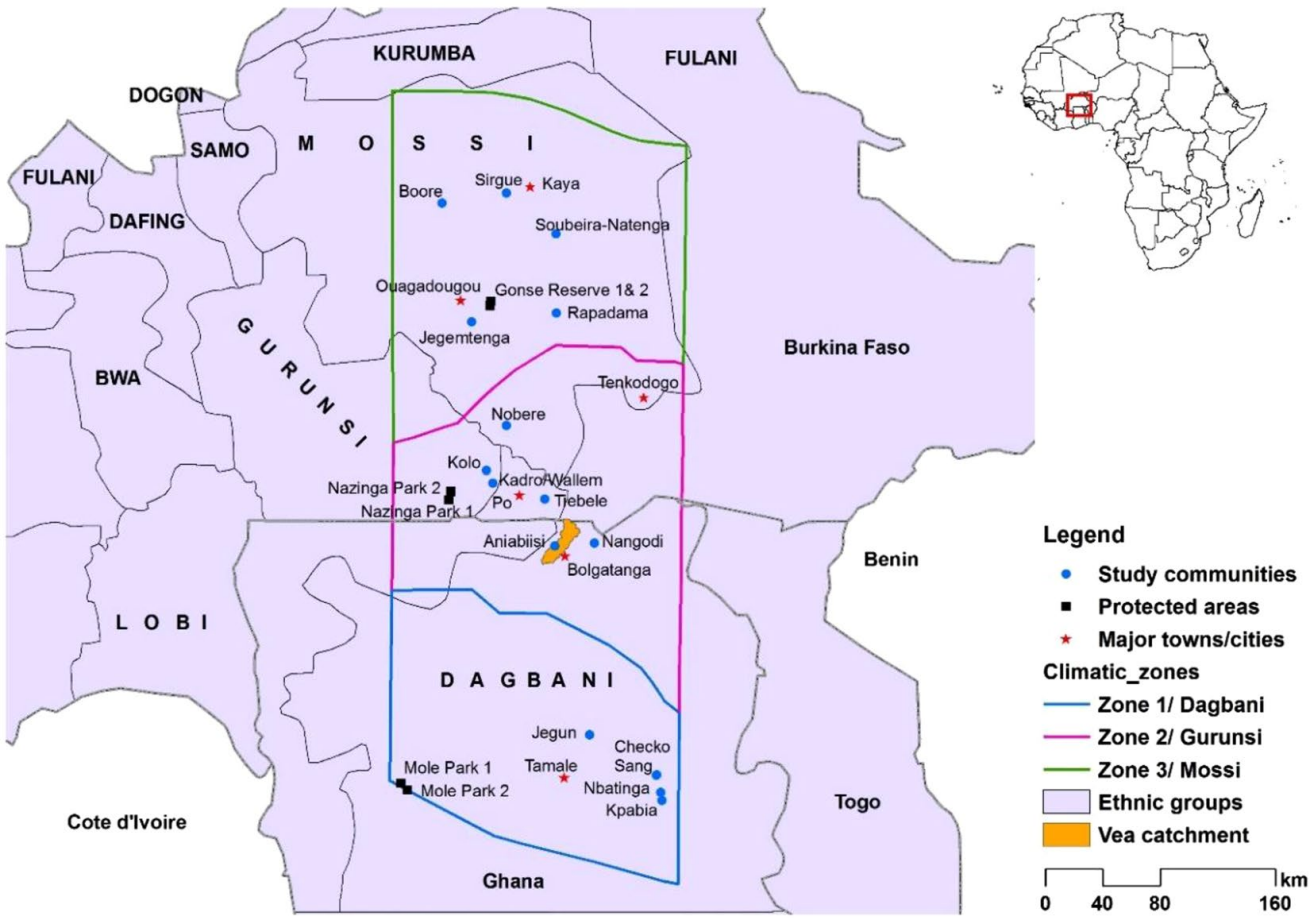
(Naah & Braun) Map depicting study areas/villages situated in northern Ghana and south-central Burkina Faso comprising three major ethnic groups (Dagbani, Gurunsi and Mossi) across a gradient of climatic aridity within the West African Sudanian savanna.

The climate of the study areas is seasonal in nature. The climatic conditions in the villages of Burkina Faso are largely semi-arid, while the study sites in Ghana vary from dry sub-humid to humid in the Northern Region and show semi-arid conditions in the Upper East Region (Fig. 1). Regarding the vegetation structure, both countries are characterized by a similarly open dry savanna type^23^. The species composition is made of various kinds of tree and grass species. The sparse tree layer is composed of economic trees/shrubs as well as other woody species which serve as good source of valuable feed for cattle, goats and sheep^24^. Common herbaceous species in both Ghana and Burkina Faso include *Loudetia togoensis, Dactyloctenium aegyptium*, *Digitaria horizontalis, Andropogon* spp, *Rottboellia cochinchinensis* (Lour.) W. D. Clayton, *Sida acuta* Burm. F. As members of predominantly farming-oriented communities, the local agro-pastoralists involve in subsistence agriculture, including traditional crops like millet, sorghum, maize and cowpea being the main crop plants.

Based on the FAO/World Bank farming systems typology, the main type of livestock system found in the semiarid areas of West Africa (and also in eastern Africa) is classified as agro-pastoral farming system^25^. Local farmers in the study areas are agro-pastoralists: they don’t only engage in crop farming but also rear various kinds of livestock in their homes such as cattle, sheep, goats and poultry (Table 1). Among these four types of livestock, cattle production is the lowest in Ghana although the trend of production has increased over the years^26^. In Burkina Faso, cattle production is also lower than goats and sheep^27^ but still higher than that of Ghana. Although other livestock types such as pigs, camels and horses are also reared in the study areas, this research only focused on forage resources used by cattle, goats and sheep. The study areas are inhabited by three dominant ethnic groups: the Mossi group in Burkina Faso, the Gurunsi (Frafra, Kasena and Nabit) on both sides of the border, and the Dagbani ethnic group predominantly living in northern Ghana (Fig. 1).

**Table 1:** Brief background information on villages (study sites) in northern Ghana and southern-central Burkina Faso.

Country	Villages (sites)	District/Province	Ethnic group	Sample size	Main landuse types & other remarks
Ghana	Sang	Mion	Dagbani	30	**Cropland**: Sorghum, maize, rice, beans, Bambara beans, cassava, yams, plantain, tomatoes, okro. **Husbandry**: Cattle, goats, sheep & poultry. **Compound farming**: No farming around houses, while goats and sheep are left free. **Other remarks**: The Dagbani people are mostly Muslims and thus don’t rear pigs. Natural vegetation has a lot of trees and land is moist & other places are humid.
	Jegun	Savelugu-Nanton	Dagbani	30	
	Cheko	Tamale Metroplis	Dagbani	30	
	Nbatinga	Mion	Dagbani	30	
	Kpabia	Mion	Dagbani	30	
Burkina Faso	Aniabiisi	Bolga Municipal	Gurunsi	76	**Cropland**: Sorghum, maize, rice, beans, Bambara beans, cassava, yams, plantain, tomatoes, okro. **Husbandry**: Cattle, goats, sheep, poultry, **Compound farming**: Commonly practiced & animals tethered during farming season. **Other remarks**: The Gurunsi people are mostly Christians and thus rear pigs. Note: Compound farming is practiced in Aniabiisi & Nagodi but not in Wallem, Kolo & Tiebele. Natural vegetation is open and relatively dry
	Nangodi	Nabdam	Gurunsi	30	
	Wallem/Kadro	Po	Gurunsi	30	
	Kolo	Jaro	Gurunsi	30	
	Tiebele	Po	Gurunsi	30	
	Nobere	Manga	Mossi	30	**Cropland**: Sorghum, maize, rice, beans, Bambara beans, cassava, yams, plantain, tomatoes, okro. **Husbandry**: Cattle, goats, sheep, poultry. **Compound farming**: No farming around houses, while goats and sheep are left free. **Other remarks**: The Mossi people are mostly Christians & traditionalists and thus rear pigs too. Natural vegetation is open with few trees and land mostly dry.
	Jegemtenga	Ouagadougou	Mossi	30	
	Rapadama	Ouagadougou	Mossi	30	
	Boore	Yaku	Mossi	30	
	Sirgui	Kaya	Mossi	30	
	Soubeira-Natenga	Kaya	Mossi	30	

### Survey design

This study was a part of a multilateral project called West African Science Service for Climate and Adapted Landuse (WASCAL) between Germany and ten West African countries (Phase One from 2012 to 2016). We administered structured ethnobotanical questionnaires to local agro-pastoralists by applying a stratified random sampling design (using ethnicity, gender and age) to collect representative data on considered ecological variables in the selected sixteen study sites (Fig. 1). LEK-related studies usually target household heads who are deemed to be have more information and leave out less influential social groups such as young boys and girls and women in the primary data gathering process^28^. However, this study tries to overcome this limitation by covering a broad spectrum of these varied social groups. For age-based stratification, we used age class definitions (e.g. such as young, middle-aged, and old adults) from a previous study in West Africa’s Sudanian savannas^29^. Field research was undertaken between October and December 2012 and from July to October 2013. In sum, we interviewed over 500 local agro-pastoralists in 16 rural communities situated within northern Ghana and southern-central Burkina Faso (Fig. 1).

### Sampling procedures for obtaining ecological information from agro-pastoralists

We validated our structured questionnaires prior to testing them on study participants. The validation process involved two local participants including a male and female in Aniabiisi village to ensure that the questionnaires were appropriate and capable of addressing all relevant aspects of the study. Based on the citation of forage resources by the local agro-pastoralists via a free list technique^23^, crucially important ecological parameters such as habitat types, current abundance, trends in abundance and ecological drivers to changes of forage resources available in local landscapes were recorded. In ethnobotanical studies, voucher reference samples are often used to aid interviews using questionnaires^30^. However, we did not use visual stimuli methods such as dry plant specimens or photos to elicit answers from local agro-pastoralists. This is because we used an unrestricted free list approach to allow local agro-pastoralists to exhibit their knowledge in forage species and other vital ecological variables. Plant specimens were gathered from herbaceous, woody and crop-related forage species cited during our fieldwork and later used to support the scientific nomenclature of these forage plants in the herbarium of the University of Ouagadougou, Burkina Faso. Reading the list of cited forage plants back to them during the ethnobotanical interviews, the local agro-pastoralists were specifically asked to indicate where each forage species was found in their environs, be it highland, lowland or both types of landscapes.

With respect to the abundance of the cited forage species, we asked local agro-pastoralists to indicate whether the cited forage species were many, few or rare within their vicinity at the time of the interviews. It has been reported by other researchers that local pastoral farmers commonly use terms like increasing, decreasing or not changing in assessment of species trends^31^. Thus, for each species mentioned, we asked them to tell us whether they were: (i) many (common), (ii) few (not common) or (iii) rare (very difficult to find). Regarding the changing trends of abundance of these forage species, the local agro-pastoralists were asked to provide information on whether the forage plant communities have changed over the past years or not. We specifically asked the local agro-pastoralists to indicate whether the trends of changes in the abundance of forage plants have increased rapidly, increased gradually, remained stable, decreased gradually or decreased rapidly.

To extract more information, we also asked the local people to provide possible reasons why they thought that their forage resources were perceived to be either increasing, decreasing or remain stable over the years. This was done to better understand local ecological drivers to changing quantity and/or quality of forage resources experienced by these local agro-pastoralists in the face of global environmental change. Finally, they were asked to describe their own solutions or management strategies with regards to the current abundance trends situation of their forage resources. To ensure the independence of individual views expressed by local agro-pastoralists during the fieldwork, we separately interviewed them to avoid exchange of ideas and answers of questions asked in each village visited. This was done by performing interviews with only one person at a time. We then informed them not to share questions and answers with yet-to-be-interviewed participants and this was strictly followed throughout the interview process. All questions were translated from English into local dialects of various ethnic groups (Dagbani, Mossi and Gurunsi) and answers provided by local agro-pastoralists in vernacular were directly translated and written down in English on the structured questionnaires (Table S1). Transcription of interviews was not needed subsequently because all answers provided by the local agro-pastoralists in their local dialects were not solely recorded in audio format but rather written down directly in English.

### Data analysis

We estimated the diversity of scientifically identified forage species in the studied communities using four diversity indices: species richness (S), species evenness (E), Shannon-Wiener diversity index (H’) and Simpson’s diversity index (SDI). For S, it represents the number of forage species via the ethnobotanical interviews in a village irrespective of whether a species is abundant or not, as also done by Zerbo *et al*.^7^. Thus, S = N, where N = number of species per village. With respective to species evenness (E), it is the ratio of the Shannon Wiener index to the natural log of S. Thus, E = H’/In (S). This reflects the balance in the distribution of individual forage species cited by local agro-pastoralists in the context of this study, which is independent of the frequency of species occurring in interviews in a village. When E is close to zero, a forage species is very similar while a value of one indicates a rather different forage species composition^7^. Regarding Shannon Wiener’s diversity Index (H’) estimation, it is also known as alpha diversity and defined as follows: H’ = −Sum (Pi*In(P*i*)), where P*i* is the proportion of individuals belonging to the i^th^ forage species (relative abundance of forage species) per village. As for Simpson’s diversity index (SDI), is also known as the Beta diversity index, whereby it is mathematically represented as: SDI = Sum (P*i*^2^). It is important to note that, species which could not be identified scientifically but were only known by their vernacular names, representing six percent were excluded from further analysis. As investigating the causalities of the ecological variables is not a focus of this study, we conducted bivariate correlation analysis to determine relationships between the diversity metrics of these forage species reported by the local agro-pastoralists in West Africa’s savannas. Given the lack of normality in some of the variables and coupled with the relatively small sample size used in this correlation analysis, we employed Kendall’s tau, a non-parametric correlation which is a better estimate of the correlation in the sampling population instead of the popularly used Spearman’s rho statistic^32^. We additionally conducted bootstrapping to obtain robust confidence intervals (CIs). The significance level for correlation coefficients was assessed at P < 0.05.

To explore perceived ecological drivers responsible for changes in abundance trends of forage plants and local conservation measures, we performed cognitive salience index (CSI) calculations for the responses provided by the local agro-pastoralists. According to Sutrop^33^, CSI is the ratio of frequency (F) to the average position (mP) and sample size (N). The CSIs were calculated based on the unrestricted responses from local agro-pastoralists and were assumed to be “free lists” for cited ecological drivers and local conservation measures. The CSI values for the 10 most important items considered in the calculation were finally visualized using pie and bar charts. The data analyses were quantitatively analyzed using the Statistical Package for the Social Sciences (SPSS) vs. 23^32^ and ANTHROPAC 4.0^34^ statistical softwares.

### Ethical approval and informed consent

Prior to the implementation of our structured questionnaires (Table S1), the research plan and ethical aspects of the interview process were approved by Department of Ecology and Natural Resources at Center for Development Research (ZEF), University of Bonn, Germany. This Department does not specifically deal with ethical issues but rather approves research projects and ensures that ethical standards are followed. In all villages visited in both Ghana and Burkina Faso, we obtained verbal permissions from the local government authorities called local District Assemblies via assemblymen and traditional chiefs, who endorsed our research questions before they were implemented (Table S1). Where necessary, we had to verbally seek for permission from the household heads to interview other members of their households for purposes of cultural sensitivities and appropriateness in such traditional communities. After getting approval from the local authorities, verbal consents were also sought from individual agro-pastoralists in all interviews across all villages in Ghana and Burkina Faso. The purpose of our research and how the data would be used for academic purposes were explicitly explained to them and assured all of them anonymity. No specific permission was required for specimen collection, as no endangered or protected species were present in communal grazing areas visited. As explained above, the ethical approval was not subject to an Institutional Review Board (IRB) prior to implementation of interviews.

## Results

### Forage species diversity from citations of local agro-pastoralists

Out of a total of 8,881 citations from local agro-pastoralists in sampled rural communities, 8,323 could be identified to scientific species. For known forage species cited, we recorded 194 different forage species, belonging to 151 genera and 52 families (see Table 2) for family richness. The species composition included grasses/herbs, leaves of trees and fresh crops including crop residues used by domestic livestock. These different forage species belong to various plant families such as Poaceae (37 forage species), Fabaceae (34) and Malvaceae (11), which were most dominant in the studied villages (Table 2). Our results also revealed that, among the ten most dominant forage species in each village analyzed, cereals such as Guinea corn (*Sorghum bicolor*), Maize (*Zea mays*) and Pearl Millet (*Pennisetum glaucum*) were the topmost crop species while legumes like Pigeon Pea (*Cajanus cajan*) and Cowpea (*Vigna unguiculata*) were top legumes grown in Ghana and Burkina Faso. *Pennisetum pedicellatum* and *Rottboellia cochinchinensis* were found to be the most frequently cited herbaceous forage species in both countries. Our findings also showed that local agro-pastoralists regarded leaves of *Afzelia africana* and *Pterocarpus erinaceus* as very important forage sources for livestock feeding in both countries. *Acacia* species such as *Acacia sieberiana*, *Faidherbia albida* and *Acacia dudgeonii* were found to be predominantly mentioned among local agro-pastoralists in Burkina Faso and in the Upper East region of study area within Ghana. The *Acacia* spp and *Balanites Egyptiaca*, which are morphologically thorny in nature, were commonly cited as good sources of forage resources for livestock grazing especially in central Burkina Faso (mostly inhabited by Mossi people) but were not highly considered for forage resources in northern Ghana (where mostly Dagbanis and Gurunsis dwell in). It was also observed that yams (*Dioscorea cayensis*) are cultivated among the Dagbanis in northern Ghana and their peels were only cited as good forage sources in this part of the study region (Fig. S1 with radar diagrams of 10 dominant forage species for all 16 villages).

**Table 2:** Family richness of forage species based on local agro-pastoralists’ citations in the study region.

No	Family name	Species richness	Percentage
1	Poaceae	37	19.07
2	Fabaceae	34	17.53
3	Malvaceae	11	5.67
4	Combretaceae	8	4.12
5	Rubiaceae	8	4.12
6	Cyperaceae	7	3.61
7	Euphorbiaceae	7	3.61
8	Moraceae	6	3.09
9	Amaranthaceae	5	2.58
10	Anacardiaceae	5	2.58
11	Asteraceae	4	2.06
12	Capparaceae	4	2.06
13	Meliaceae	4	2.06
14	Bombacaceae	3	1.55
15	Lamiaceae	3	1.55
16	Rutaceae	3	1.55
17	Solanaceae	3	1.55
18	Commelinaceae	2	1.03
19	Convolvulaceae	2	1.03
20	Cucurbitaceae	2	1.03
21	Musaceae	2	1.03
22	Pedaliaceae	2	1.03
23	Sterculiaceae	2	1.03
24	Verbenaceae	2	1.03
25	Acanthaceae	1	0.52
26	Aizoaceae	1	0.52
27	Annonaceae	1	0.52
28	Apocynaceae	1	0.52
29	Arecaceae	1	0.52
30	Asclepiadaceae	1	0.52
31	Balanitaceae	1	0.52
32	Bignoniaceae	1	0.52
33	Cannabaceae	1	0.52
34	Caricaceae	1	0.52
35	Celastraceae	1	0.52
36	Dioscoceaceae	1	0.52
37	Ebenaceae	1	0.52
38	Icacinaceae	1	0.52
39	Loganiaceae	1	0.52
40	Loranthaceae	1	0.52
41	Lythraceae	1	0.52
42	Moringaceae	1	0.52
43	Myrtaceae	1	0.52
44	Nyctaginaceae	1	0.52
45	Olacaceae	1	0.52
46	Opiliaceae	1	0.52
47	Polygalaceae	1	0.52
48	Rhamnaceae	1	0.52
49	Sapindaceae	1	0.52
50	Sapotaceae	1	0.52
51	Scrophulariaceae	1	0.52
52	Simaroubaceae	1	0.52
	Total	194	100

For relationships among biodiversity metrics, it was evident that forage species richness was significantly correlated with the Shannon Wiener diversity index and Simpson’s diversity index; while it has an insignificant negative relationship with forage species evenness (Table 3). Also, forage species evenness was significantly related to Simpson’s diversity index but insignificantly correlated with the Shannon Wiener diversity index (Table 3). It was also revealed that the Shannon Wiener diversity index had a highly significant relationship with Simpson’s diversity index (Table 3).

**Table 3:** Correlation matrix illustrating various species diversity metrics for forage species cited by local agro-pastoralists resident in varied rural communities in northern Ghana and southern-central Burkina Faso.

Diversity metrics	Species richness	Species evenness	Shannon’s index	Simpson’s index
Species richness	1	−0.019 [−0.393, 0.452]	0.735** [0.379, 0.950]	0.502* [0.136, 0.749]
Species evenness	16	1	0.327 [−0.212, 0.775]	0.549* [0.133, 0.838]
Shannon’s index	16	16	1	0.775** [0.402, 0.891]
Simpson’s index	16	16	16	1

Bias correlated and accelerated bootstrap (BCa) 95 percent CIs reported in brackets.

Unless otherwise stated, bootstrap results are based on 1000 samples. Note: N = Sample size = 16 villages, ns = not significant (P > 0.05), *P < 0.05, **P < 0.01, ***P < 0.001.

### Habitat types, abundance and changing trends of forage resources availability from perceptions of local agro-pastoralists

**Figure 2:**
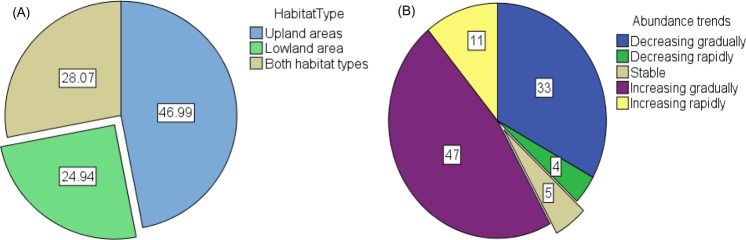
(Naah & Braun) Pie charts depicting proportions of local agro-pastoralists’ perceptions based on (**A**) Habitat types for cited forage species, and (**B**) Abundance trends of forage species.

The local agro-pastoralists exhibited extensive knowledge and understanding of the habitat distribution of cited forage resources available in their local landscapes. The geography of the study areas is largely undulating (ups and downs), but not mountainous in nature. The habitat types associated with forage species cited by local agro-pastoralists were mostly uplands/highlands, lowlands or both types of landscapes. It was also recorded that some forage species, especially many trees, could exist in both lowland and upland areas. Our results indicated that most cited forage species were believed to be associated with upland/highland habitats (constituting 47 percent), as compared to 25 percent for lowland areas and 28 percent considered to be suitable for both habitat types (Fig. 2A).

**Figure 3:**
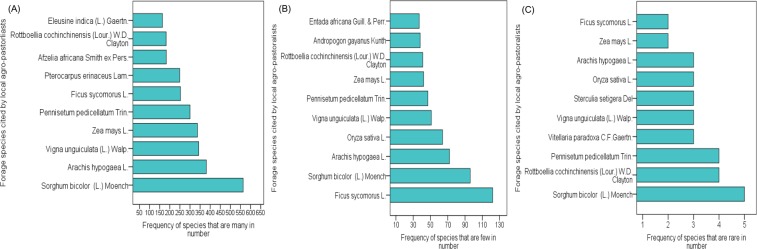
(Naah & Braun) Local agro-pastoralists’ perception on the abundance levels of ten most frequently cited forage species for: (**A**) Many forage species, (**B**) Few forage species, and (**C**) Rare forage species.

With respect to forage species abundance for livestock grazing, local agro-pastoralists exhibited different views on whether a species is very common, less common or very difficult to find in their environs. About 82 percent of all cited species by local agro-pastoralists can considered to be commonly available in their landscape. About 17 percent of cited species were said to be only few in number with respect to their availability while one percent of citations were reported to be rare. For instance, crop-related forage sources such as cereals (*S. bicolor* and *Z. mays*), and legumes (Groundnuts-*Arachis hypogaea* and *V. unguiculata*) were regarded common by local agro-pastoralists while herbaceous and woody forage species were perceived to be less common. *Ficus sycomorus* was reported as one forage species which topped those considered to be fewer in number. It is important to state that local agro-pastoralists’ assessment of the abundance of forage species was quite ambiguous as some of these cited forage species included *S. bicolor*, *A. hypogaea*, *R. cochinchinensis* and *P. pedicellatum* were cited to be many, few or rare based on individual experiences (Fig. 3A–C).

Furthermore, it was confirmed that there were changes in abundance trends of forage resources over the past few years. Local agro-pastoralists stated that about 47 percent of forage species cited were perceived to have a gradually increasing trend, while about 33 percent of reported forage species were believed to be gradually decreasing in number. Additionally, about 11 percent and four percent of the reported items were said to be rapidly increasing, rapidly decreasing and stable in population over the years respectively (Fig. 2B).

### Local ecological drivers responsible for changes in abundance trends of forage plants and local conservation measures

**Figure 4:**
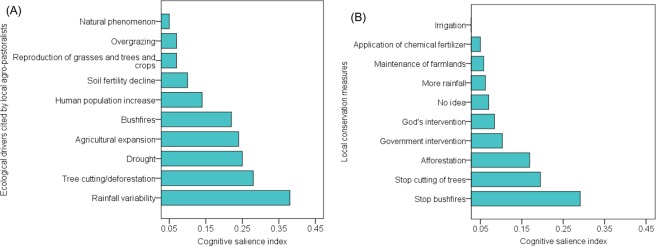
(Naah & Braun) Bar charts illustrating the cultural importance with Cognitive Salience Indexes (CSI values) for topmost (**A**) Local ecological drivers influencing abundance trends of forage species according to local agro-pastoralists, and (**B**) Local conservation measures of forage resources according to agro-pastoralists.

A wide array of ecological drivers believed to be responsible for changing trends in the abundance of forage resources were provided by the local agro-pastoralists. Ecological threats negatively affect the production and availability of crop-related as well as naturally available woody and herbaceous forage resources. According to local agro-pastoralists, rainfall variability (e.g. late rains or disproportionate down pour of rains within a short period of time) is the most important driver to either a rapidly or slowly declining trend in crop production also leading to lesser quantities of crop residues for livestock grazing. Related to rainfall variability are drought spells, which local farmers experience from time to time. Drought incidences were widely reported as very serious threats particularly in Burkina Faso which lead to declining trends in the availability of forage resources. Another dominant reason mentioned by local agro-pastoralists why there are declining crop-related forage resources was soil fertility deterioration which negatively affects crop production (Fig. 4A). It was also intimated that many local farmers should practice crop rotational farming e.g. *S. bicolor* vs soybeans (*Glycline max*) to control the presence of witchweed (*Striga* spp).

Regarding the availability of tree species used as livestock feed, deforestation (trees cutting), agricultural expansion, human population growth and bushfires were perceived by local agro-pastoralists to be highly salient ecological drivers to changes in the abundance of woody forage resources (Fig. 4A). All these drivers are human-induced for various latent reasons. Also, it is evidently clear that the herbaceous population serves as a primary source of feed for domestic livestock in an open savanna vegetation. The local agro-pastoralists recognized the fact that bushfires and overgrazing leads to a rapid or gradual decline of herbaceous forage species for livestock grazing in the sampled rural communities in both countries (Fig. 4A). The various changes in the abundance of forage species were also attributed to natural phenomena such as germination, natural death, and so on (Fig. 4A). Our study also revealed that local agro-pastoralists were quite aware of their own actions that could influence the changing abundance of natural forage resources. Moreover, the local agro-pastoralists suggested means through which their rich LEK could be applied in traditional management and the regulation of forage plants.

According to local agro-pastoralists, the declining trend in the abundance of crop residues for provision of livestock feed could be arrested via several ways. On the part of rainfall variability, this is nothing that can be done by the local agro-pastoralists since that is a natural phenomenon and requires God’s intervention as they reported. However, irrigation was mentioned to be an intervening practice that could help with respect to rainfall variability, but rural communities lack the resources (e.g. financial and physical) to make irrigation possible. Chemical fertilizers (and compost manure) are applied to deal with the widespread decline of natural soil fertility. The local agro-pastoralists also indicated that they needed to ensure high maintenance of their farmlands via regular weeding. Proper conditions in their farms would lead to increased production of food crops as well as crop residues for domestic livestock feed (Fig. 4B).

Furthermore, it was suggested by local agro-pastoralists that cutting down of trees (e.g. mangoes, oranges, shea nuts, etc.) for charcoal production and fuel wood should be stopped, while afforestation of useful trees should be encouraged by fellow farmers to increase the frequency of rainfall incidences and increase soil fertility. Considering the dangers of indiscriminate bush burning inherent in the study region it was also suggested that bushfires should either be avoided completely, or early bush burning be done to curtail intensive destruction of available dead and living forage materials. Others even added that culprits when caught burning the environment for whatever reason should be severely punished as a deterrent to others (Fig. 4B). There should be moderate use of herbicides as well as avoidance of overgrazing by livestock should be encouraged although it might be difficult to get a consensus on that, as claimed by some agro-pastoralists. The local people believed public sensitization/education on the consequences of bushfires (destruction of soils, grasses and trees), herbicides and tree cutting should be conducted preferably by agricultural extension officers. Additionally, creation of alternative economic opportunities via government pro-poor interventions such as fertilizer subsidies, farm implements (tractors), accessible funds (money) and so on to illiterate-dominated rural communities for poverty alleviations and livelihood improvement. Regarding the issue of inter-annual rainfall variability, some local agro-pastoralists said that they needed to pray to God or make animal sacrifices to small gods (ancestors) for more rainfall since no one has control over changing rainfall patterns (Fig. 4B).

## Discussion

### Estimation of forage species diversity from local agro-pastoralists’ LEK

The most represented plant families (Table 2) in the recall of local agro-pastoralists consisted of Poaceae, Fabaceae and Malvaceae, as similarly reported in the study area by Zizka *et al*.^35^. The most dominant genera of forage species cited included the *Acacia*, *Ficus* and *Andropogon*. This could be indicative of the predominant role local climatic conditions play in the distributional patterns of forage-related LEK among local agro-pastoralists^23^. In a semi-arid savanna ecosystem in South Africa, it was also found that local agro-pastoralists had extensive ecological knowledge on the grass compositions in three different regions in Botswana^36^.

For estimation of forage species diversity and other biodiversity metrics using information gathered from local agro-pastoralists, it was found that species richness had a negative correlation with species evenness. This finding supports Zhang *et al*.^37^, who found species richness and evenness to be negatively correlated in their study of meadow communities on the eastern Qinghai-Tibetan Plateau in China. The overall relationship between richness and evenness has been found to be contradictory. Existing studies suggest a positive^38^ or a strongly negative correlation^39^ between richness and evenness, or they see them as largely independent^40^ which is often associated with plant communities and difficult to be easily reconciled^37^. This may also be explained by the vegetation dynamics of the forage plants pertaining to various villages located in both Burkina Faso and Ghana (Fig. S1). Strong correlations between diversity measures should not be surprising as they represent aspects of the same phenomenon^41^.

### Assessment of habitat types, abundance and trends of forage resources availability from perspectives of local agro-pastoralists

Responses from local agro-pastoralists on cited forage species vary with respect to habitat distributions. This was expected as LEK is unevenly distributed among its holders^42,43^. Many cited forage species were associated with upland areas. For instance, crop species (cereal and legumes) were linked to upland areas, except rice which is mostly grown in lowland areas. Especially tree species were cited to exist in both upland and lowland areas. These findings reflect how local people hold different opinions regarding where to suitably find forage plants based on their personal life experience.

The findings of this study revealed that about 82 percent of citations were regarded by local agro-pastoralists as many in their landscapes, while the rest of them were either few or rare in number. This reflects the availability of various forage resources for feeding their livestock. There was, however, ambiguity in the assessment of abundance of forage species by local agro-pastoralists which may be attributable to their individual viewpoint. Also, competing uses of crops cultivated in the study areas for both humans and livestock consumption might have influenced how they assessed their abundance. For woody and herbaceous forage species, it may dependent upon an agro-pastoralists’ knowledge and interest in these forage species. Our findings are supported by studies which suggested that LEK is not only unique to different communities^44^ but also differs significantly from an individual to another even within the same community^36^.

With respect to the abundance trends of forage resources assessed by local agro-pastoralists, our findings reflect a positive assessment. Most local agro-pastoralists held the view that many forage species (57 percent) cited were either gradually or rapidly increasing. We also found that other cited forage species either had a gradually or rapidly decreasing trend, which is strongly linked to various drivers such as overgrazing or deforestation. For forage species which showed a stable trend, some local agro-pastoralists felt that herbaceous forage species are always available, and they are therefore neither declining nor increasing trend over the years (Fig. 2B).

### Investigation of local ecological drivers and local conservation issues

Ecological drivers responsible for the changing trends in the abundance of forage resources (as explained above) are of utmost importance for subsistence farming activities in the study areas. According to our findings, rainfall variability is perceived as the most important driver to changing trends of forage resources, particularly crop-related forage. This may be because surveyed local people are mainly subsistence farmers and therefore depend majorly upon rain-fed agriculture. Thus, when annual rainfall is very low, crop production becomes poor leading to unavailability of crop residues to support livestock feed. This suggests that fresh crop plants and crop residues availability is strongly modulated by rainfall variability, as good rains would lead to better yields. Also, rains enable growth of fresh and more grasses/forbs for livestock grazing. Also related to rainfall variability is the incidence of droughts, which is detrimental to farming activities. The issue of drought is more often experienced by local agro-pastoralists living close to the north of Burkina Faso as compared to northern Ghana, where rains are more reliable. The problems of rainfall variability and grazing pressure are also reported elsewhere in Africa^45,46^. Deforestation is the second most important driver to vegetation dynamics in the study areas. People cut down trees for firewood and charcoal production. Also, with the quest to undertake agricultural expansion to meeting growing demands for more food and feed, uneconomic trees will have to be slashed and burnt. As a result, leaves of trees may not be available for feeding livestock especially during dry seasons. Bushfires and overgrazing incidences are also rampant in the surveyed communities (Fig. 2B, Table S2). Our findings are supported by literature, which indicates that threats to the abundance of woody vegetation and forage plant species are a combination of anthropogenic and natural causal factors such as agriculture, overgrazing, deforestation, bushfires, droughts and so on^12,47^.

When asked about how above-stated ecological drivers could be curbed, an array of conservation measures was given. Stoppage of deforestation and avoiding bushfires by all local farmers topped the solutions offered. It was also suggested that tree populations should be replenished via afforestation. Government and or NGOs interventions were seriously needed to overcome problems of droughts as well as for the provision of support for chemical fertilizers and irrigational facilities to improve farming (Fig. 4B, Table S3). It is thus amply evident that indigenous management strategies employed by local agro-pastoralists may ensure efficient utilization and form the basis for decision making processing to conserve grazing lands and forage resources for livestock production amidst climate change and variability in the research region^12,48^.

## Conclusions and Recommendations

In this study, ecological variables such as forage species diversity, their habitat distribution, abundance trends as well as associated ecological drivers in terms of livestock production were assessed from the viewpoints of local agro-pastoralists in rural West Africa. This study confirms an intimate interaction among humans, domestic livestock and ecosystem services (e.g. forage provisioning) from the natural environment to sustainably serve the needs of domestic livestock and their owners. It also becomes clear that the biodiversity metrics estimated from the information provided by local agro-pastoralists are diverse and widely distributed within the topography of the study areas. For instance, about 50 percent of cited forage species are perceived to be associated with upland areas as compared to low areas and both habitat types combined. Regarding the dynamics of abundance of forage plants over the past few years, most of the forage species are still believed to be increasing gradually. This reflects a positive opinion of forage plant population status as compared to other negative trends cited by local agro-pastoralists. These varying trends in changing abundance of forage species are greatly influenced by underlying ecological drivers including rainfall variability, deforestation for charcoal production and farming activities, drought and bushfires, and livestock grazing. Since local agro-pastoralists’ livelihoods are heavily dependent upon natural resource availability in their communal lands, they are well aware of the need to manage their forage resources sustainably. This is especially true with regards to their own proposed local solutions including avoidance of bushfires (or encouraging early bushfires), moderately use herbicides, application of organic manure/chemical fertilizer, crop rotational and shifting cultivation. It should be noted that local farmers often know very well which specific ecological variables are the most relevant for their livestock production and their views should be considered in land management strategies and/or policies. For instance, indigenous knowledge in biological control of witchweed (*Striga* spp) to increase cereals production (including crop residues) by intercropping cereals with e.g. *Glycine max* in the study areas is more potent and environmentally-friendly than use of weedicides. Also, organic farming practices by local farmers such as use of compost manure, non-destruction of economic woody species on farms and limited clearing of farmlands support biodiversity conservation unlike establishment of plantations. Notwithstanding, these local efforts are often negatively affected by direct climate change impacts coupled with environmental degradation. Thus, Ghana government agricultural policy of planting for food and jobs should make good use of local agricultural knowledge. We therefore conclude that the findings of this study have management and policy relevance since knowledge and experiences of smallholder farmers could be harnessed to effectively address food and nutrition insecurity issues particularly in developing countries amidst global warming.

## Supplementary information

Supplementary information files

## Data Availability

The datasets used and/or analyzed during the current study are available from the corresponding author on reasonable request. However, all relevant data from this study are included in this article and (and its Supplementary Information files).
