# Supplementary material for: Local agro-pastoralists’ perspectives on forage species diversity, habitat distributions, abundance trends and ecological drivers for sustainable livestock production in West Africa
Source: Sci Rep. 2019 Feb 8;9:1707. doi: 10.1038/s41598-019-38636-1 (PMC6368564; doi:10.1038/s41598-019-38636-1)
Supplement: Supplementary file 1 — Supplementary information files [file 41598_2019_38636_MOESM1_ESM.pdf]

Local agro-pastoralists' perspectives on forage species diversity, habitat distributions, abundance trends and ecological drivers for sustainable livestock production in West Africa

John-Baptist S. N. Naah* and Boris Braun

*Corresponding author: E-mail address: jeanlebaptist@yahoo.co.uk (J-B. S. N. Naah)

Fig. S1: Summary of the ten most dominant forage species composition given by local agro-pastoralists located in individual studied rural communities located in both Ghana and Burkina Faso.

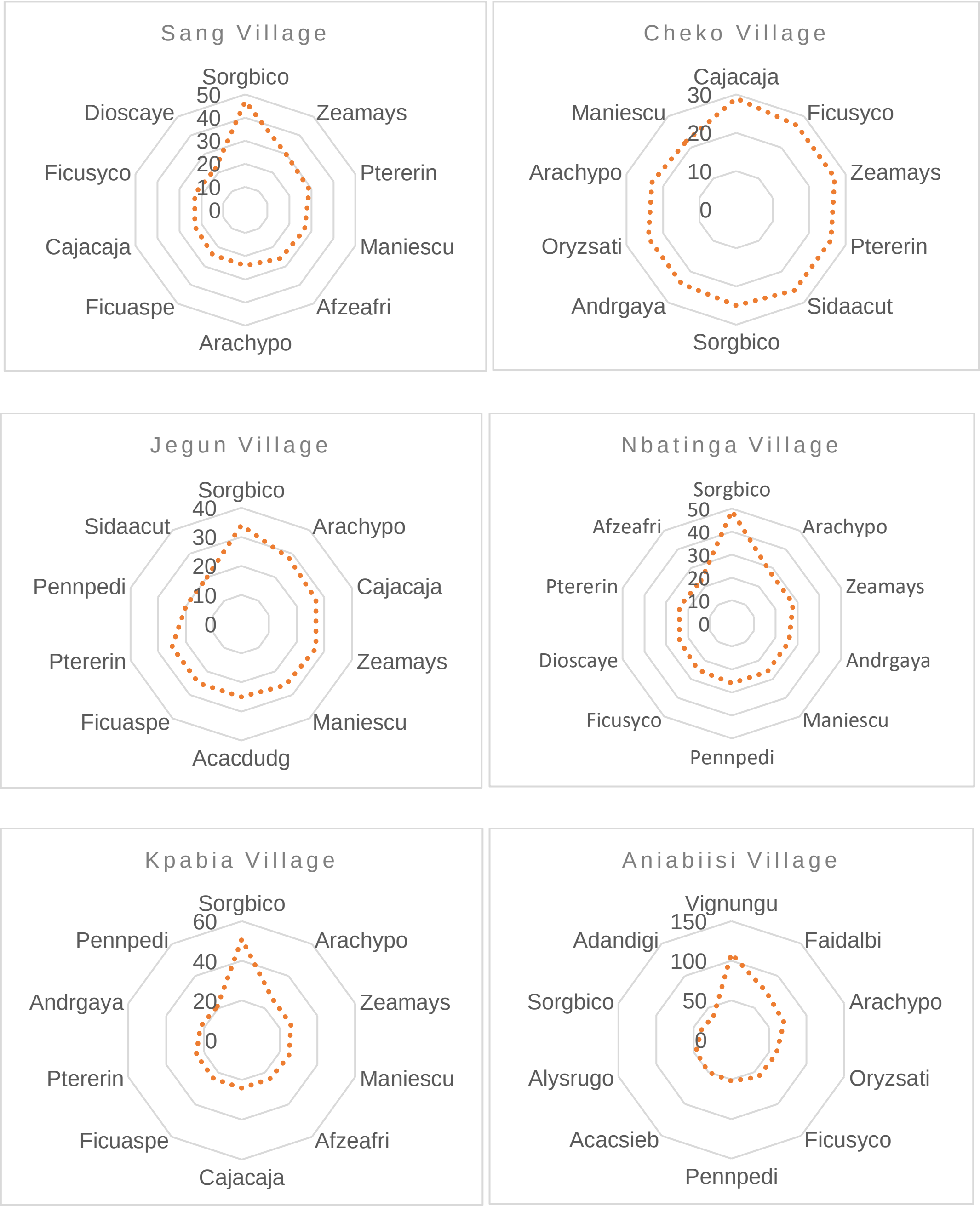

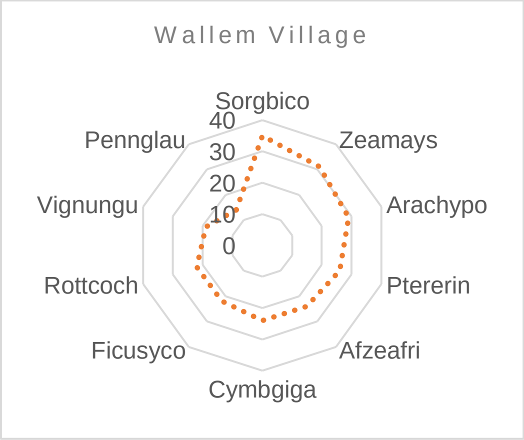
Wallem Village
Sorgbico
Zeamays
Arachypo
Ptererin
Afzeafri
Cymbgiga
Ficusyco
Rottcoch
Vignungu
Pennglau
40
30
20
10
0

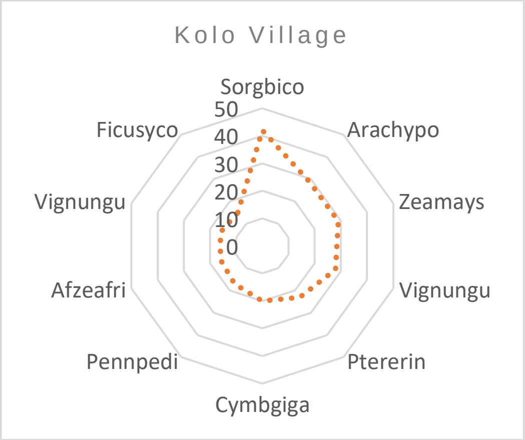
Kolo Village
Sorgbico
Arachypo
Zeamays
Vignungu
Ptererin
Cymbgiga
Pennpedi
Afzeafri
Vignungu
Ficusyco
50
40
30
20
10
0

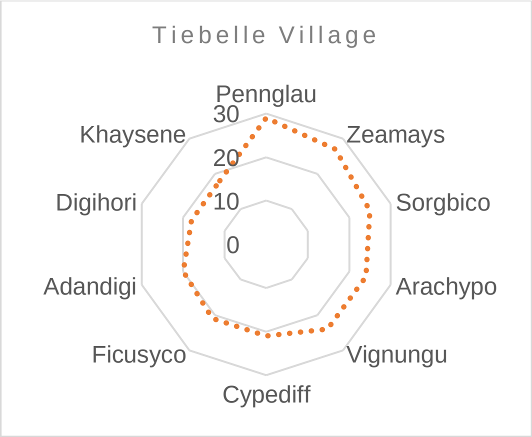
Tiebelle Village
Pennglau
Zeamays
Sorgbico
Arachypo
Vignungu
Cypediff
Ficusyco
Adandigi
Digihori
Khaysene
30
20
10
0

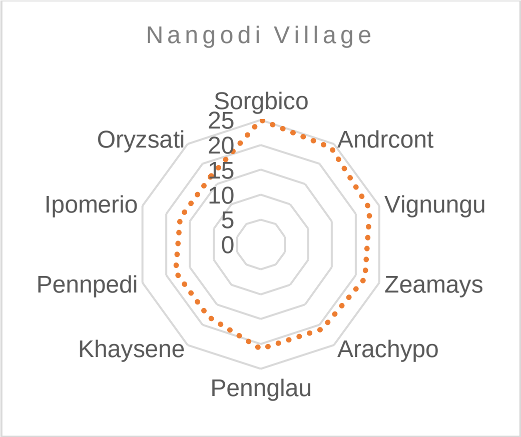
Nangodi Village
Sorgbico
Andrcont
Vignungu
Zeamays
Arachypo
Pennglau
Khaysene
Pennpedi
Ipomerio
Oryzsati
25
20
15
10
5
0

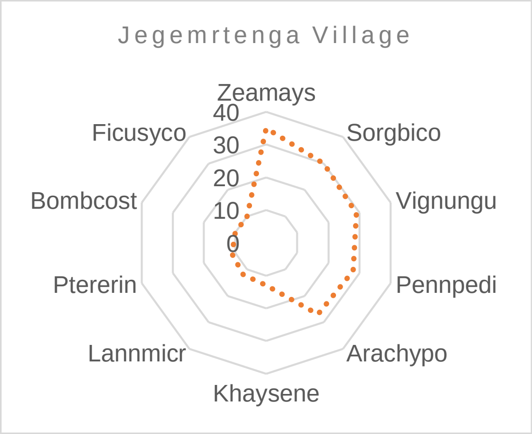
Jegemrtenga Village
Zeamays
Sorgbico
Vignungu
Pennpedi
Arachypo
Khaysene
Lannmicr
Ptererin
Bombcost
Ficusyco
40
30
20
10
0

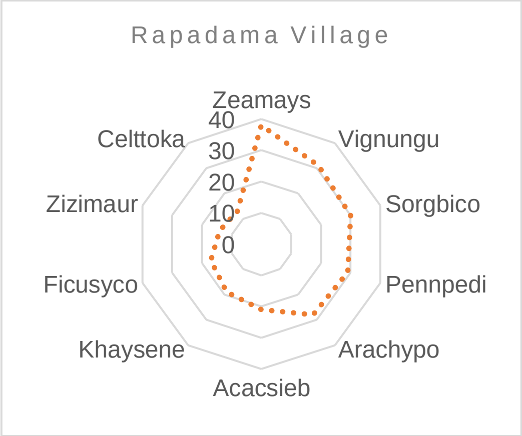
Rapadama Village
Zeamays
Vignungu
Sorgbico
Pennpedi
Arachypo
Acacsieb
Khaysene
Ficusyco
Zizimaur
Celttoka
40
30
20
10
0

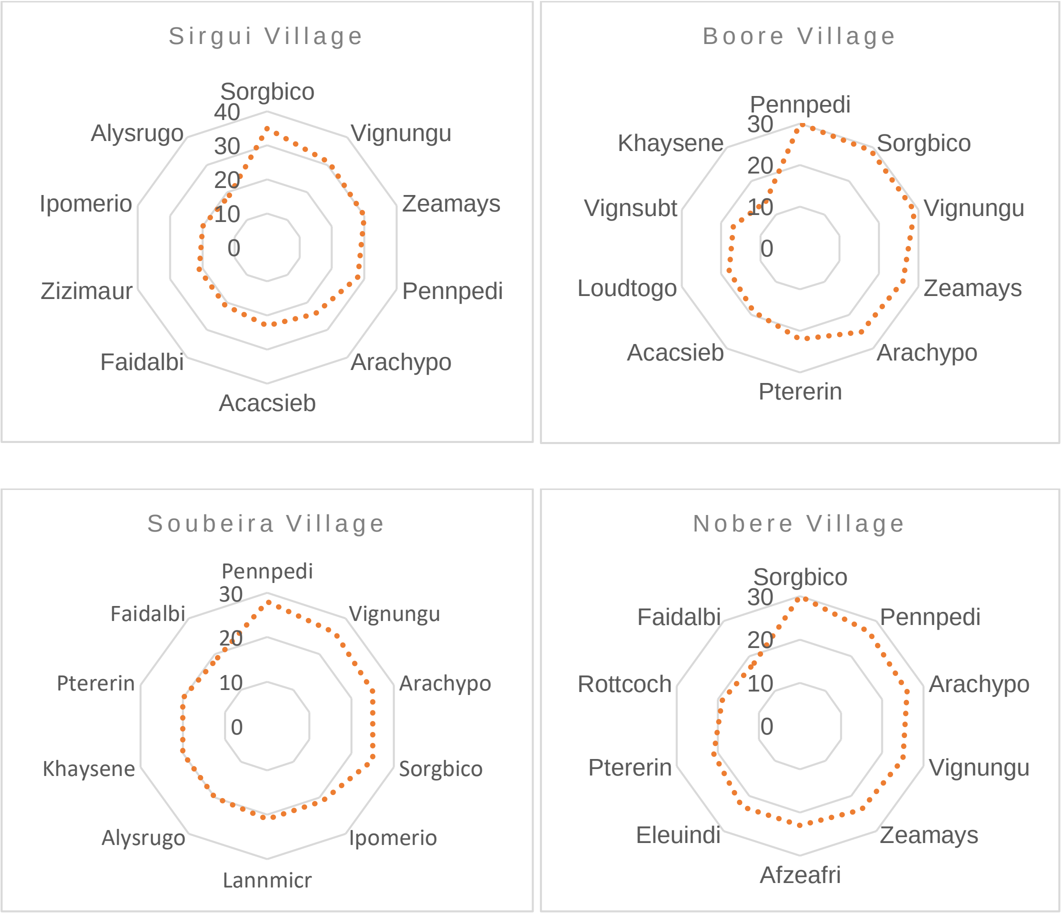

Note: Adandigi=*Adansonia digitata* L., Acacdudg=*Acacia dudgeonii* Craib ex Holland, Acacsieb=*Acacia sieberiana* DC., Afzeafri=*Afzelia africana* Smith ex Pers., Arachypo=*Arachis hypogaea* L., Alysrugo=*Alysicarpus rugosus* (Willd.) DC., Andrcont=*Andropogon contortus* L., Andrgaya=*Andropogon gayanus* Kunth, Bombcost=*Bombax costatum* Pellegr. & Vuill., Cajacaja=*Cajanus cajan* (L.) Millsp., Cymb.giga= *Cymbopogon giganteus* Chiov., Cypediff=*Cyperus difformis* L., Digihori=*Digitaria horizontalis* Willdenow, Dioscaye=*Dioscorea cayenensis* Lam., Eleuindi=*Eleusine indica* (L.) Gaertn, Faidalbi=*Faidherbia albida* (Del.), Ficuaspe=*Ficus aspera*, Ficusyco=*Ficus sycomorus* L., Khaysene=*Khaya senegalensis* (Desr.) A. Juss., Lannmicr=*Lannea microcarpa* Engl. & K. Krause, Loudtogo=*Loudetia togoensis* (Pilg.) C. E. Hubb., Ipomerio= *Ipomoea eriocarpa* R. Br., Maniescu=*Manihot esculenta* Crantz, Oryzsati=*Oryza sativa* L., Pennglau=*Pennisetum glaucum* L., Pennpedi=*Pennisetum pedicellatum* Trin, Ptererin=*Pterocarpus erinaceus* Lam., Rottcoch=*Rottboellia cochinchinensis* (Lour.) W. D. Clayton, Sidaacut=*Sida acuta* Burm. F., Sorgbico=*Sorghum bicolor* (L.) Moench, Vignungu=*Vigna unguiculata* (L.) Walp, Vignsubt=*Vigna subterranea* (L.) Verdc., Zeamays=*Zea mays* L. and Zizimari=*Ziziphus Mauritania* Lam.

Table S1: Structured questionnaire for performance of individual surveys for obtaining alternative information from local agro-pastoralists on LEK distributional patterns, valaution criteria and local perception forage species diversity in for both Ghana and Burkina Faso.

General introduction: This structured questionnaire is designed to understand, document and assess valuable ethnobotanical knowledge on available forage resources by local agro-pastoralists ('local experts') in above-mentioned West African countries. Respondents are assured that all information being gathered from them is exclusively used for academic purposes and would be treated as confidential and not used with ill intent. Answers given in vernacular by participants were directly transcribed into English from the translators of the interviews.

Section I: Demographic profile of respondents

1.1 Name of respondent (optional)				
1.2 Name of community (location)				
1.3 Age/age class (in years)				
1.4 Gender	Male		Female	
1.5 Ethnicity	Dagbani	Mossi	Gurunsi	
1.6 Educational level	No school	Primary	Secondary	Tertiary
1.7 Religion	Christian	Muslim	Traditional	Atheist
1.8 Household size				
1.9 Residential status	Native	Migrant settler	Nomadic	
1.10Date of interview (DD/MM/YYYY)				
1.11 Name of interviewer				

Section I: Implicit ranking based on free-list of forage resources

Intro: I will write down your responses and later ask you further questions about a list of plants that you will tell me!

Q1: Please name as many as possible plants and/or crop residues which are important for feeding cattle, goats and sheep in your vicinity!	
Free list of fodder plants	Free list continues

Section II: Ecological parameters (habitat types and dominance/abundance of fodder plants)

i. Habitat types

Q2: Where are these above-mentioned fodder plants and/or crop residues found within your landscape?		
Lowland (L)	Highland/Upland (H)	Other habitat-please specify

ii. Dominance/abundance

Q3: What do you say about the number or population size of these preferred fodder plants and/or crop residues for your 3 livestock types (dominance/abundance)?		
Many (common)	Few (not common)	Rare or locally extinct

Section III: Changes over time: Trends and drivers

i. Trends

Q4: What do you say about the number or population size of these preferred fodder plants and/or crop residues for your 3 livestock types (dominance/abundance)?		
Decreasing (D) trend	Increasing (I) trend	Stable (S) trend

ii. Ecological divers

Q5: What do you think are the possible causes responsible for the changing population size of these named fodder plants and/or crop residues? (Please write down the answer in keywords/short sentences)
a.
b.
c.
d.

iii. The way forward

Q6: What do you think can be done individually and collectively to handle such changes in fodder plants and/or crop residues numbers? (Please write down the answer in keywords/short sentences)
a.
b.
c.
d.

The END!

Thank you very much for your time!

Table S2: Perceived ecological drivers and their calculated cognitive salience indices in the study areas (DOCX).

No	Perceived ecological drivers	Salience
1	Rainfall variability	0.375
2	Tree cutting/deforestation	0.276
3	Drought	0.250
4	Agricultural expansion	0.235
5	Bushfires	0.221
6	Human population increase	0.144
7	Soil fertility decline	0.096
8	Overgrazing	0.073
9	Reproduction of grasses and trees and crops	0.072
10	Natural phenomenon	0.049
11	Animal population increase	0.038
12	Firewood/charcoal production	0.025
13	Continuous farming/weeding	0.019
14	Infrastructural development (houses)	0.015
15	Destruction of trees/crops by elephants &other animals	0.014
16	Poverty	0.011
17	Destruction of plants by wind	0.011
18	Scarcity of farming lands	0.010
19	High temperatures (too much sun)	0.008
20	We don’t plant more trees	0.005
21	Lack of alternative economic ventures	0.004
22	Establishment of forest reserve	0.004
23	No more many farmers interested	0.004
24	Grasses are naturally occurring grasses (need no planting)	0.004
25	Tree pruning	0.003
26	Weeding (some plants like weeding)	0.003
27	Some grasses kill others	0.003
28	Appearance of different grass species	0.003
29	Bad methods of farming	0.003
30	No interest in growing some particular plants	0.002
31	Rocky topography	0.002
32	Transportation difficulties due to bad roads	0.002
33	Labor cost of bringing feeding home	0.002
34	Neglect of customs with ‘tendaana’ (landlord)	0.002
35	More trees and grasses negatively affect crops growth	0.002
36	Endemic species	0.001
37	Floods	0.001
38	Migration of other tribes like Mossi, Fulanis to this place	0.001
39	Crops needs a lot of watering & buying from agric officers	0.001
40	Disappear when people step on it	0.001
41	People from town come to cut a lot of forage resources	0.000
42	Crop rotation	0.000
43	Absence of horses	0.000

44	Tubers are difficult to handle but dont need a lot of sun	0.000
45	Animals feed on them much	0.000
46	Plant diseases-"kongsi" (leprocy)	0.000
47	Non-development of science	0.000
48	Hunger and need to earn money	0.000

Table S3: Local conservation measures and their calculated cognitive salience indices in the study areas (DOCX).

No	Local conservation measures	Salience
1	Stop bushfires	0.291
2	Stop cutting of trees	0.195
3	Afforestation	0.169
4	Government intervention	0.103
5	God's intervention	0.084
6	No idea	0.070
7	More rainfall	0.062
8	Maintenance of farmlands	0.058
9	Application of chemical fertilizer	0.050
10	Irrigation	0.026
11	Growing more crops	0.025
12	Public education	0.022
13	Stop/moderate use of weedicides	0.019
14	Early bushfires	0.016
15	Fallow management/shifting cultivation	0.013
16	Decrease the number of animals by selling some	0.013
17	People should obey customs in the village	0.012
18	Application of organic manure	0.012
19	Proper storage of crop residues	0.012
20	Establishment of watchdog committees to fight bushfires	0.010
21	Balance between grazing land and cropland proportions	0.008
22	Crop rotational farming	0.008
23	Tithering of livestock in farming season	0.007
24	Pruning the trees	0.005
25	Stop overgrazing of some grasses	0.004
26	Stop Fulanis from migrating to our village with many cows	0.004
27	Community assistance	0.002
28	Change of scattered housing setup	0.002
29	No clear regulation on use of lands for grazing	0.002
30	Chiefs intervention	0.002
31	Early sowing of crops	0.002
32	Praying for more money	0.002
33	Stop compound farming	0.001
34	Animals should be allowed to feed on grasses	0.001
35	Migration of strangers	0.001
36	Funeral performance should not be delayed	0.001
37	Protection of grasses and trees for livestock	0.001

Text S1: Free list raw data the informants and listed 'items' or way forward are regarded as free list items to obtain values of salience (DOCX).

i) Free list of ecological drivers
#DSAMO01
Rainfall variability
Reproduction of grasses and trees and crops
Agricultural expansion
Soil fertility decline
#DSAMO02
Agricultural expansion
Bushfires
Tree cutting/deforestation
#DSAMO03
Agricultural expansion
Bushfires
Human population increase
No employment opportunities
Infrastructural development(houses)
#DSAMO04
Agricultural expansion
Bushfires
Human population increase
#DSAMO05
Agricultural expansion
Bushfires
Human population increase
Reproduction of grasses and trees and crops
#DSAFI06
Agricultural expansion
Human population increase
Reproduction of grasses and trees and crops
Tree cutting/deforestation
Rainfall variability
Infrastructural development(houses)
#DSAFI07
Rainfall variability
No more many farmers interested
Tree cutting/deforestation
Agricultural expansion
Bushfires
#DSAFO08
No more many farmers interested
Good soils
Human population increase
Transplanting and planting of trees
#DSAFY09
Good soils

Human population increase
Grasses are naturally occurring grasses(need no planting)
Crops needs alot of watering & buying from agric officers
No more many farmers interested
#DSAMI10
Grasses are naturally occurring grasses(need no planting)
Rainfall variability
#DSAMY11
Rainfall variability
Drought
Overgrazing
#DSAMI12
Overgrazing
Bushfires
Drought
#DSAMY13
Bushfires
Drought
Rainfall variability
Rocky topography
#DSAMI14
Rainfall variability
Rocky topography
Use of chemicals to spray grasses
Weeding (some plants like weeding)
Labour cost of bringing feeding home
Transportation difficulties due to bad roads
General financial problems
#DSAMY15
Rainfall variability
Use of chemicals to spray grasses
Weeding (some plants like weeding)
Labour cost of bringing feeding home
Transportation difficulties due to bad roads
General financial problems
Disappear when people step on it
Bushfires
Human population increase
No employment opportunities
#DSAMI16
Bushfires
Rainfall variability
Human population increase
No employment opportunities
Agricultural expansion
#DSAMY17
Bushfires
Human population increase

Agricultural expansion
Rainfall variability
Use of chemicals to spray grasses
#DSAMY18
Bushfires
Rainfall variability
Use of chemicals to spray grasses
Wet and dry soil types
#DSAMI19
Rainfall variability
Wet and dry soil types
Drought
Firewood/charcoal production
Use of chemicals to spray grasses
#DSAFI20
Drought
Human population increase
Firewood/charcoal production
Use of chemicals to spray grasses
Tree cutting/deforestation
Agricultural expansion
#DSAFO21
Agricultural expansion
Rainfall variability
#DSAFI22
Rainfall variability
Continuous farming/weeding
Soil fertility decline
#DSAFO23
Rainfall variability
Continuous farming/weeding
Soil fertility decline
Bushfires
#DSAFY24
Bushfires
Soil fertility decline
#DSAFY25
Soil fertility decline
Tree cutting/deforestation
Natural phenomenon
Firewood/charcoal production
#DSAFI26
Natural phenomenon
Tree cutting/deforestation
Firewood/charcoal production
Bushfires
#DSAFO27
Bushfires

Firewood/charcoal production
Tree cutting/deforestation
#DSAFY28
Bushfires
Tree cutting/deforestation
Despite bush burning, plants grow fast and increase in numbers
#DSAFY29
Despite bush burning, plants grow fast and increase in numbers
Reproduction of grasses and trees and crops
#DSAFO30
Reproduction of grasses and trees and crops
#DCHMY01
Reproduction of grasses and trees and crops
Agricultural expansion
Rainfall variability
#DCHMO02
Agricultural expansion
Rainfall variability
Drought
#DCHMI03
Drought
Rainfall variability
Human population increase
Animal population increase
Use of chemicals to spray grasses
Rainfall variability
#DCHMI04
Rainfall variability
Human population increase
Animal population increase
Use of chemicals to spray grasses
Rainfall variability
#DCHMY05
Rainfall variability
Human population increase
Animal population increase
Use of chemicals to spray grasses
Bushfires
#DCHMO06
Bushfires
Rainfall variability
Agricultural expansion
Human population increase
Animal population increase
Tree cutting/deforestation
Firewood/charcoal production
#DCHMI07
Agricultural expansion

Human population increase
Animal population increase
Bushfires
Soil fertility decline
#DCHMO08
Bushfires
Soil fertility decline
Human population increase
#DCHMY09
Human population increase
Animal population increase
Soil fertility decline
#DCHMY10
Human population increase
Animal population increase
Soil fertility decline
Bushfires
#DCHMY11
Bushfires
Soil fertility decline
Agricultural expansion
#DCHFO12
Agricultural expansion
Rainfall variability
Soil fertility decline
Natural phenomenon
#DCHMI13
Rainfall variability
Soil fertility decline
Natural phenomenon
Grasses are naturally occurring grasses(need no planting)
Plants/Grasses are no use to humans beings
Tree cutting/deforestation
Agricultural expansion
#DCHFI14
Tree cutting/deforestation
Agricultural expansion
Firewood/charcoal production
Reproduction of grasses and trees and crops
#DCHFY15
Agricultural expansion
Reproduction of grasses and trees and crops
Rainfall variability
#DCHMO16
Rainfall variability
Reproduction of grasses and trees and crops
Agricultural expansion
Soil fertility decline

Tree cutting/deforestation
Bushfires
#DCHMO17
Agricultural expansion
Soil fertility decline
Planting or protection of economic or very useful trees
#DCHMI18
Agricultural expansion
Reproduction of grasses and trees and crops
Tree cutting/deforestation
#DCHFO19
Tree cutting/deforestation
Rainfall variability
#DCHFO20
Rainfall variability
Agricultural expansion
Reproduction of grasses and trees and crops
Tree cutting/deforestation
#DCHFO21
Agricultural expansion
Reproduction of grasses and trees and crops
Tree cutting/deforestation
#DCHFI22
Tree cutting/deforestation
Firewood/charcoal production
#DCHFI23
Tree cutting/deforestation
Firewood/charcoal production
Human population increase
#DCHFO24
Human population increase
Tree cutting/deforestation
Firewood/charcoal production
#DCHFI25
Tree cutting/deforestation
Firewood/charcoal production
#DCHFY26
Tree cutting/deforestation
Firewood/charcoal production
Lack of alternative economic ventures
#DCHFY27
Tree cutting/deforestation
Firewood/charcoal production
Natural phenomenon
Reproduction of grasses and trees and crops
#DCHFY28
Natural phenomenon
Reproduction of grasses and trees and crops

Tree cutting/deforestation
Firewood/charcoal production
#DCHFI29
Tree cutting/deforestation
Firewood/charcoal production
Lack of alternative economic ventures
#DCHFY30
Firewood/charcoal production
Lack of alternative economic ventures
Bushfires
Tree cutting/deforestation
#DJEMO01
Bushfires
Tree cutting/deforestation
Firewood/charcoal production
Human population increase
Soil fertility decline
Scarcity of farming lands
#DJEMY02
Human population increase
Soil fertility decline
Scarcity of farming lands
Rainfall variability
Reproduction of grasses and trees and crops
Use of chemicals to spray grasses
#DJEMY03
Rainfall variability
Reproduction of grasses and trees and crops
Soil fertility decline
Agricultural expansion
More trees and grasses negatively affect crops growth
#DJEMI04
Rainfall variability
Reproduction of grasses and trees and crops
More trees and grasses negatively affect crops growth
Bushfires
Tree cutting/deforestation
Agricultural expansion
Human population increase
Animal population increase
#DJEMY05
Agricultural expansion
Bushfires
Human population increase
Animal population increase
Reproduction of grasses and trees and crops
Tree cutting/deforestation
Firewood/charcoal production

Planting or protection of economic or very useful trees
#DJEMY06
Bushfires
Agricultural expansion
Human population increase
Animal population increase
Reproduction of grasses and trees and crops
Planting or protection of economic or very useful trees
Tree cutting/deforestation
Hunger and need to earn money
#DJEMO07
Agricultural expansion
Tree cutting/deforestation
Human population increase
#DJEMO08
Bushfires
Human population increase
Agricultural expansion
Use of chemicals to spray grasses
Economic trees are not destroyed
#DJEMO09
Agricultural expansion
Use of chemicals to spray grasses
Economic trees are not destroyed
Tree cutting/deforestation
Firewood/charcoal production
Bushfires
Soil fertility decline
Overgrazing
Reproduction of grasses and trees and crops
Human population increase
Animal population increase
People from town come to cut a lot of foragge resources
#DJEMO10
Agricultural expansion
Tree cutting/deforestation
Firewood/charcoal production
Bushfires
Soil fertility decline
Overgrazing
Reproduction of grasses and trees and crops
Human population increase
Animal population increase
People from town come to cut a lot of foragge resources
#DJEFY11
Bushfires
Reproduction of grasses and trees and crops
Tree cutting/deforestation

Overgrazing
Agricultural expansion
Planting or protection of economic or very useful trees
#DJEFY12
Agricultural expansion
Planting or protection of economic or very useful trees
#DJEFI13
Planting or protection of economic or very useful trees
Agricultural expansion
Tree cutting/deforestation
Firewood/charcoal production
Tubers are difficult to handle but dont need alot of sun
#DJEFI14
Agricultural expansion
Rainfall variability
Plants/Grasses are no use to humans beings
Reproduction of grasses and trees and crops
#DJEFO15
Rainfall variability
Plants/Grasses are no use to humans beings
Reproduction of grasses and trees and crops
Use of chemicals to spray grasses
Continuous farming/weeding
#DJEFY16
Use of chemicals to spray grasses
Continuous farming/weeding
Agricultural expansion
Reproduction of grasses and trees and crops
#DJEFY17
Agricultural expansion
Reproduction of grasses and trees and crops
#DJEFI18
Reproduction of grasses and trees and crops
Tree cutting/deforestation
Agricultural expansion
#DJEMY19
Tree cutting/deforestation
Rainfall variability
Soil fertility decline
Firewood/charcoal production
#DJEMY20
Tree cutting/deforestation
Use of chemicals to spray grasses
We dont plant more trees
Poverty
#DJEMY21
We dont plant more trees
Poverty

Rainfall variability
#DJEFY22
Rainfall variability
Tree cutting/deforestation
We dont plant more trees
#DJEFO23
Tree cutting/deforestation
We dont plant more trees
Bushfires
#DJEFY24
Bushfires
Agricultural expansion
Human population increase
Soil fertility decline
Destruction of plants by wind
#DJEMI25
Agricultural expansion
Human population increase
Soil fertility decline
Destruction of plants by wind
Bushfires
Overgrazing
Continuous farming/weeding
Tree cutting/deforestation
#DJEMI26
Tree cutting/deforestation
Bushfires
Continuous farming/weeding
Overgrazing
Firewood/charcoal production
#DJEFO27
Bushfires
Overgrazing
Reproduction of grasses and trees and crops
#DJEFI28
Reproduction of grasses and trees and crops
Natural phenomenon
#DJEFO29
Natural phenomenon
#DJEFO30
Natural phenomenon
Rainfall variability
#DNBMY01
Rainfall variability
Agricultural expansion
Human population increase
Reproduction of grasses and trees and crops
#DNBMI02

Reproduction of grasses and trees and crops
Agricultural expansion
Human population increase
Natural phenomenon
Rainfall variability
Bushfires
#DNBMI03
Natural phenomenon
Rainfall variability
Bushfires
Human population increase
Reproduction of grasses and trees and crops
Agricultural expansion
Use of chemicals to spray grasses
Tree cutting/deforestation
Firewood/charcoal production
#DNBMI04
Agricultural expansion
Use of chemicals to spray grasses
Human population increase
Rainfall variability
Animal population increase
Animals feed on them much
#DNBMI05
Rainfall variability
Animal population increase
Human population increase
Overgrazing
Use of chemicals to spray grasses
Agricultural expansion
Plant diseases -"Kongsi" (leprocy)
#DNBMO06
Agricultural expansion
Bushfires
Reproduction of grasses and trees and crops
Human population increase
#DNBMO07
Rainfall variability
Reproduction of grasses and trees and crops
Human population increase
Good soils
Agricultural expansion
Bushfires
#DNBMY08
Good soils
Human population increase
Agricultural expansion
Use of chemicals to spray grasses

Bushfires
Rainfall variability
#DNBMO09
Agricultural expansion
Use of chemicals to spray grasses
Bushfires
Rainfall variability
Chemical fertilizer use
Drought
Human population increase
#DNBMO10
Chemical fertilizer use
Rainfall variability
Drought
Human population increase
Agricultural expansion
Natural phenomenon
Bushfires
#DNBMO11
Agricultural expansion
Human population increase
Natural phenomenon
Chemical fertilizer use
Rainfall variability
Bushfires
Tree cutting/deforestation
#DNBMI12
Agricultural expansion
Bushfires
Human population increase
Tree cutting/deforestation
Planting or protection of economic or very useful trees
#DNBFY13
Planting or protection of economic or very useful trees
Natural phenomenon
Rainfall variability
Soil fertility decline
Tree cutting/deforestation
Firewood/charcoal production
#DNBFO14
Tree cutting/deforestation
Firewood/charcoal production
Natural phenomenon
Rainfall variability
Soil fertility decline
Bushfires
Human population increase
Animal population increase

Planting or protection of economic or very useful trees
Overgrazing
Use of chemicals to spray grasses
Crop rotation
Agricultural expansion
#DNBFI15
Bushfires
Human population increase
Animal population increase
Rainfall variability
Natural phenomenon
Reproduction of grasses and trees and crops
#DNBFY16
Natural phenomenon
Reproduction of grasses and trees and crops
Human population increase
#DNBFI17
Reproduction of grasses and trees and crops
Human population increase
Drought
Use of chemicals to spray grasses
#DNBFO18
Drought
Use of chemicals to spray grasses
Rainfall variability
Soil fertility decline
#DNBFI19
Rainfall variability
Soil fertility decline
Scarcity of farming lands
#DNBFO20
Rainfall variability
Scarcity of farming lands
Continuous farming/weeding
#DNBFI21
Continuous farming/weeding
Rainfall variability
Bushfires
Soil fertility decline
#DNBFI22
Bushfires
Soil fertility decline
Rainfall variability
Overgrazing
#DNBFY23
Rainfall variability
Overgrazing
Planting or protection of economic or very useful trees

#DNBFY24
Rainfall variability
#DNBFO25
Rainfall variability
Some grasses kill others e.g Bulaasani kills Tantee
#DNBFO26
Some grasses kill others e.g Bulaasani kills Tantee
Tree cutting/deforestation
Tree prunning
#DNBFY27
Tree prunning
Rainfall variability
#DNBMY28
Rainfall variability
Bushfires
Drought
#DNBMY29
Bushfires
Drought
Agricultural expansion
Soil fertility decline
#DNBMY30
Agricultural expansion
Soil fertility decline
Tree cutting/deforestation
#DKPMI01
Tree cutting/deforestation
Rainfall variability
Agricultural expansion
Use of chemicals to spray grasses
Plants/Grasses are no use to humans beings
#DKPMY02
Rainfall variability
Agricultural expansion
Use of chemicals to spray grasses
Tree cutting/deforestation
Bushfires
Soil fertility decline
#DKPMY03
Bushfires
Agricultural expansion
Soil fertility decline
Tree cutting/deforestation
Use of chemicals to spray grasses
Planting or protection of economic or very useful trees
#DKPFY04
Bushfires
Agricultural expansion

Use of chemicals to spray grasses
Rainfall variability
Tree cutting/deforestation
#DKPFY05
Rainfall variability
Economic trees are not destroyed
Soil fertility decline
Bushfires
Agricultural expansion
#DKPFO06
Soil fertility decline
Rainfall variability
Bushfires
Agricultural expansion
Natural phenomenon
Reproduction of grasses and trees and crops
#DKPFO07
Natural phenomenon
Agricultural expansion
Reproduction of grasses and trees and crops
#DKPFO08
Natural phenomenon
Agricultural expansion
Reproduction of grasses and trees and crops
Rainfall variability
#DKPFI09
Natural phenomenon
Reproduction of grasses and trees and crops
Rainfall variability
Agricultural expansion
Tree cutting/deforestation
Bushfires
#DKPFO10
Tree cutting/deforestation
Bushfires
Agricultural expansion
Rainfall variability
Soil fertility decline
Natural phenomenon
#DKPMY11
Rainfall variability
Soil fertility decline
Natural phenomenon
Bushfires
Agricultural expansion
Human population increase
#DKPMO12
Human population increase

Agricultural expansion
Overgrazing
#DKPMI13
Human population increase
Overgrazing
Agricultural expansion
Use of chemicals to spray grasses
Continuous farming/weeding
#DKPMI14
Use of chemicals to spray grasses
Agricultural expansion
Continuous farming/weeding
Bushfires
#DKPFI15
Bushfires
Use of chemicals to spray grasses
Agricultural expansion
#DKPMO16
Agricultural expansion
Bushfires
#DKPMO17
Bushfires
Agricultural expansion
#DKPMY18
Agricultural expansion
Natural phenomenon
#DKPMI19
Natural phenomenon
Overgrazing
Rainfall variability
#DKPMI20
Bushfires
Agricultural expansion
Natural phenomenon
#DKPMY21
Bushfires
Agricultural expansion
Natural phenomenon
#DKPMO22
Bushfires
Agricultural expansion
Natural phenomenon
Continuous farming/weeding
#DKPMO23
Bushfires
Agricultural expansion
Continuous farming/weeding
#DKPMO24

Agricultural expansion
Rainfall variability
Overgrazing
Use of chemicals to spray grasses
#DKPFY25
Rainfall variability
Overgrazing
Use of chemicals to spray grasses
#DKPFO26
Rainfall variability
#DKPFI27
Rainfall variability
#DKPFI28
Rainfall variability
Wind distruction
Tree cutting/deforestation
#DKPFY29
Wind distruction
Tree cutting/deforestation
Rainfall variability
#DKPFY30
Rainfall variability
Overgrazing
Destruction of plants by wind
#GANFY01
Destruction of plants by wind
Rainfall variability
Agricultural expansion
#GANMO02
Rainfall variability
Agricultural expansion
Tree cutting/deforestation
#GANMI03
Tree cutting/deforestation
Rainfall variability
#GANMO04
Rainfall variability
#GANMO05
Rainfall variability
Overgrazing
Tree cutting/deforestation
Absence of horses
#GANFI06
Rainfall variability
Overgrazing
Tree cutting/deforestation
Agricultural expansion
#GANMO07

Rainfall variability
Agricultural expansion
#GANFY08
Agricultural expansion
Rainfall variability
#GANMO09
Rainfall variability
Soil fertility decline
Tree cutting/deforestation
Natural phenomenon
Destruction of plants by wind
#GANFY10
Rainfall variability
Natural phenomenon
#GANFO11
Natural phenomenon
Rainfall variability
#GANFY12
Rainfall variability
Overgrazing
#GANFY13
Rainfall variability
Animals destroying crops
#GANFY14
Rainfall variability
#GANFI15
Rainfall variability
Animals destroying crops
#GANFI16
Rainfall variability
#GANMY17
Rainfall variability
Animals destroying crops
#GANMO18
Rainfall variability
Animals destroying crops
Natural phenomenon
#GANFI19
Natural phenomenon
Rainfall variability
#GANFY20
Rainfall variability
Soil fertility decline
#GANFI21
Rainfall variability
Soil fertility decline
#GANFO22
Rainfall variability

#GANFY23
Rainfall variability
Agricultural expansion
Tree cutting/deforestation
#GANFY24
Tree cutting/deforestation
Agricultural expansion
#GANFI25
Agricultural expansion
#GANMY26
Rainfall variability
Soil fertility decline
Continuous farming/weeding
#GANMY27
Rainfall variability
Soil fertility decline
Continuous farming/weeding
Agricultural expansion
#GANFY28
Agricultural expansion
Soil fertility decline
Rainfall variability
Animals destroying crops
#GANMO29
Rainfall variability
Animals destroying crops
#GANMO30
Rainfall variability
#GANFI31
Rainfall variability
#GANMI32
Rainfall variability
Overuse of useful grasses for roofing
#GANMY33
Overuse of useful grasses for roofing
Bushfires
Agricultural expansion
Tree cutting/deforestation
#GANMY34
Bushfires
Agricultural expansion
Human population increase
Infrastructural development(houses)
Farmers don’t plant more trees
#GANFY35
Human population increase
Infrastructural development(houses)
Rainfall variability

Farmers don’t plant more trees
Bushfires
#GANFI36
Rainfall variability
#GANFI37
Rainfall variability
#GANMY38
Rainfall variability
Agricultural expansion
High temperatures (too much sun)
#GANMY39
Agricultural expansion
Rainfall variability
High temperatures (too much sun)
#GANMY40
Rainfall variability
Agricultural expansion
High temperatures (too much sun)
#GANMO41
Rainfall variability
High temperatures (too much sun)
#GANMI42
Rainfall variability
Neglect of customs with Tendaana (landlord)
#GANMY43
Rainfall variability
Neglect of customs with Tendaana (landlord)
#GANMI44
Rainfall variability
#GANFI45
Rainfall variability
#GANMY46
Rainfall variability
#GANFI47
Rainfall variability
#GANMY48
Rainfall variability
#GANFY49
Rainfall variability
#GANFO50
Rainfall variability
#GANFY51
Rainfall variability
High temperatures (too much sun)
#GANFY52
Rainfall variability
#GANFY53
Rainfall variability

#GANMY54
Rainfall variability
#GANFO55
Rainfall variability
Soil fertility decline
#GANFI56
Rainfall variability
Soil fertility decline
#GANMO57
Rainfall variability
#GANFO58
Rainfall variability
#GANFO59
Rainfall variability
#GANMY60
Rainfall variability
Soil fertility decline
Continuous farming/weeding
#GANMO61
Rainfall variability
Soil fertility decline
Continuous farming/weeding
#GANMO62
Rainfall variability
#GANMI63
Rainfall variability
Bushfires
Reproduction of grasses and trees and crops
#GANFI64
Rainfall variability
Bushfires
Reproduction of grasses and trees and crops
#GANFI65
Rainfall variability
#GANFO66
Rainfall variability
Drought
Human population increase
#GANMI67
Rainfall variability
Drought
Human population increase
#GANMI68
Rainfall variability
Soil fertility decline
Tree cutting/deforestation
#GANMI69
Rainfall variability

Soil fertility decline
Tree cutting/deforestation
No financial resources to better improve farming practices
#GANFI70
Rainfall variability
No financial resources to better improve farming practices
#GANMI71
Rainfall variability
#GANMO72
Rainfall variability
#GANMO73
Rainfall variability
#GANMO74
Rainfall variability
#GANFI75
Rainfall variability
Soil fertility decline
Agricultural expansion
#GANMY76
Rainfall variability
Bad methods of farming
Overgrazing
Bushfires
Destruction of plants by wind
Floods
#GWKMI01
Rainfall variability
Bad methods of farming
Overgrazing
Bushfires
Destruction of plants by wind
Floods
#GWKMY02
Rainfall variability
#GWKMI03
Rainfall variability
Tree cutting/deforestation
#GWKMI04
Rainfall variability
#GWKMI05
Rainfall variability
Destruction of trees by elephants
#GWKMO06
Rainfall variability
Tree cutting/deforestation
#GWKMO07
Rainfall variability
Destruction of trees by elephants

#GWKMO08
Rainfall variability
#GWKFY09
Rainfall variability
Soil fertility decline
#GWKFY10
Rainfall variability
Soil fertility decline
Destruction of trees by elephants
#GWKFO11
Rainfall variability
Agricultural expansion
#GWKMI12
Agricultural expansion
Soil fertility decline
#GWKFO13
Rainfall variability
Soil fertility decline
Use of chemicals to spray grasses
Human population increase
Planting or protection of economic or very useful trees
#GWKFI14
Planting or protection of economic or very useful trees
Bushfires
Use of chemicals to spray grasses
Agricultural expansion
Tree cutting/deforestation
#GWKFO15
Tree cutting/deforestation
Bushfires
Human population increase
Rainfall variability
Planting or protection of economic or very useful trees
Destruction of trees by elephants
#GWKFI16
Rainfall variability
Chemical fertilizer use
Establishment of Forest reserve
Human population increase
#GWKFO17
Chemical fertilizer use
Human population increase
Reproduction of grasses and trees and crops
#GWKFY18
Human population increase
Reproduction of grasses and trees and crops
Rainfall variability
#GWKMY19

Reproduction of grasses and trees and crops
Rainfall variability
Planting or protection of economic or very useful trees
Human population increase
#GWKMO20
Human population increase
#GWKMY21
Human population increase
Bushfires
Natural phenomenon
Rainfall variability
#GWKFI22
Human population increase
Planting or protection of economic or very useful trees
Reproduction of grasses and trees and crops
Rainfall variability
#GWKMY23
Rainfall variability
Planting or protection of economic or very useful trees
Reproduction of grasses and trees and crops
Agricultural expansion
Rainfall variability
#GWKFI24
Agricultural expansion
Human population increase
#GWKMO25
Agricultural expansion
Rainfall variability
Human population increase
#GWKFY26
Rainfall variability
Human population increase
Tree cutting/deforestation
Bushfires
#GWKFY27
Agricultural expansion
Rainfall variability
Human population increase
Reproduction of grasses and trees and crops
#GWKFI28
Reproduction of grasses and trees and crops
Establishment of Forest reserve
Human population increase
#GWKFO29
Human population increase
Overgrazing
Use of chemicals to spray grasses
Tree cutting/deforestation

Agricultural expansion
#GWKMY30
Tree cutting/deforestation
Agricultural expansion
Bushfires
#GKOMI01
Animal population increase
Tree cutting/deforestation
Soil fertility decline
#GKOFY02
Soil fertility decline
Bushfires
Overgrazing
Animals destroying crops
#GKOFY03
Animals destroying crops
Rainfall variability
Bushfires
Overgrazing
Animal population increase
#GKOFO04
Rainfall variability
Bushfires
Overgrazing
Chemical fertilizer use
#GKOFO05
Rainfall variability
Chemical fertilizer use
Overgrazing
Animal population increase
#GKOFO06
Overgrazing
Animal population increase
Chemical fertilizer use
#GKOFI07
Animal population increase
Animals destroying crops
#GKOFI08
Animal population increase
Animals destroying crops
Rainfall variability
Bushfires
Poverty
#GKOFI09
Poverty
Bushfires
Tree cutting/deforestation
Rainfall variability

Soil fertility decline
#GKOFY10
Soil fertility decline
Rainfall variability
#GKOFY11
Rainfall variability
#GKOFI12
Rainfall variability
#GKOFO13
Rainfall variability
Planting or protection of economic or very useful trees
#GKOMY14
Rainfall variability
Planting or protection of economic or very useful trees
Chemical fertilizer use
#GKOMY15
Chemical fertilizer use
Rainfall variability
Planting or protection of economic or very useful trees
#GKOMY16
Rainfall variability
Planting or protection of economic or very useful trees
#GKOMY17
Rainfall variability
Planting or protection of economic or very useful trees
#GKOMI18
Rainfall variability
Planting or protection of economic or very useful trees
Animal population increase
Overgrazing
Tree cutting/deforestation
#GKOMO19
Tree cutting/deforestation
Human population increase
Agricultural expansion
Soil fertility decline
#GKOMO20
Human population increase
Agricultural expansion
Soil fertility decline
#GKOMO21
Human population increase
Soil fertility decline
Tree cutting/deforestation
#GKOMO22
Human population increase
Soil fertility decline
Animal population increase

Tree cutting/deforestation
Agricultural expansion
#GKOMI23
Agricultural expansion
Soil fertility decline
High temperatures (too much sun)
Rainfall variability
#GKOMI24
High temperatures (too much sun)
Rainfall variability
Migration of other tribes like mossi, fulanis to this place
Good soils
Human population increase
Animal population increase
Agricultural expansion
#GKOMO25
Agricultural expansion
Tree cutting/deforestation
#GKOFY26
Use of chemicals to spray grasses
Animal population increase
Tree cutting/deforestation
Natural phenomenon
Human population increase
Soil fertility decline
#GKOFO27
Soil fertility decline
Human population increase
Bushfires
#GKOFI28
Human population increase
Animal population increase
#GKOMI29
Animal population increase
Bushfires
Human population increase
#GKOMY30
Human population increase
Bushfires
#GTIFI01
Bushfires
Soil fertility decline
Poverty
Tree cutting/deforestation
#GTIFO02
Bushfires
Soil fertility decline
Poverty

Animal population increase
Overgrazing
Planting or protection of economic or very useful trees
Rainfall variability
#GTIFI03
Rainfall variability
Bushfires
Animal population increase
Overgrazing
Tree cutting/deforestation
#GTIFO04
Bushfires
Rainfall variability
Animal population increase
Overgrazing
Planting or protection of economic or very useful trees
Soil fertility decline
#GTIFO05
Soil fertility decline
Bushfires
Animal population increase
Tree cutting/deforestation
Rainfall variability
#GTIFY06
Rainfall variability
Planting or protection of economic or very useful trees
#GTIFO07
Rainfall variability
Bushfires
#GTIMY08
Bushfires
Rainfall variability
Planting or protection of economic or very useful trees
#GTIFI09
Planting or protection of economic or very useful trees
Rainfall variability
Tree cutting/deforestation
#GTIFI10
Rainfall variability
Bushfires
#GTIMO11
Bushfires
Rainfall variability
Soil fertility decline
#GTIMY12
Bushfires
Soil fertility decline
Rainfall variability

#GTIMY13
Rainfall variability
Bushfires
#GTIMI14
Bushfires
Rainfall variability
Good soils
#GTIMY15
Rainfall variability
Bushfires
Agricultural expansion
Good soils
Planting or protection of economic or very useful trees
#GTIMI16
Bushfires
Planting or protection of economic or very useful trees
Agricultural expansion
Good soils
Chemical fertilizer use
Tree cutting/deforestation
#GTIMI17
Tree cutting/deforestation
Bushfires
Overgrazing
Rainfall variability
#GTIMO18
Bushfires
Overgrazing
Rainfall variability
Appearance of different grass species
Planting or protection of economic or very useful trees
Improved understanding and exposure now to crop varieties
#GTIMY19
Appearance of different grass species
Planting or protection of economic or very useful trees
Improved understanding and exposure now to crop varieties
#GTIMO20
No freqquent bushfires
Rainfall variability
Improved understanding and exposure now to crop varieties
#GTIMO21
Improved understanding and exposure now to crop varieties
Rainfall variability
Bushfires
Tree cutting/deforestation
#GTIMI22
Bushfires
Tree cutting/deforestation

Animal dispersal of grasses seeds
Improved understanding and exposure now to crop varieties
#GTIFO23
Bushfires
Tree cutting/deforestation
Soil fertility decline
Reproduction of grasses and trees and crops
#GTIFY24
Bushfires
Soil fertility decline
Planting or protection of economic or very useful trees
Improved understanding and exposure now to crop varieties
#GTIFY25
Improved understanding and exposure now to crop varieties
Agricultural expansion
#GTIFY26
Agricultural expansion
Chemical fertilizer use
Human population increase
Tree cutting/deforestation
#GTIFY27
Agricultural expansion
Chemical fertilizer use
#GTIMO28
Chemical fertilizer use
Tree cutting/deforestation
Firewood/charcoal production
Human population increase
#GTIMI29
Human population increase
Agricultural expansion
Bushfires
#GTIFI30
Human population increase
Reproduction of grasses and trees and crops
Chemical fertilizer use
#GNAMO01
Human population increase
Tree cutting/deforestation
Bushfires
Natural phenomenon
Rainfall variability
#GNAMI02
Natural phenomenon
Bushfires
Reproduction of grasses and trees and crops
Rainfall variability
#GNAMI03

Rainfall variability
Human population increase
Reproduction of grasses and trees and crops
#GNAMI04
Rainfall variability
Human population increase
Reproduction of grasses and trees and crops
#GNAMI05
Reproduction of grasses and trees and crops
Agricultural expansion
Rainfall variability
Planting or protection of economic or very useful trees
Some for roofing purposes
#GNAMY06
Some for roofing purposes
Agricultural expansion
Rainfall variability
Reproduction of grasses and trees and crops
#GNAMY07
Rainfall variability
Reproduction of grasses and trees and crops
Infrastructural development(houses)
Human population increase
#GNAFI08
Human population increase
Reproduction of grasses and trees and crops
Rainfall variability
#GNAFI09
Rainfall variability
Human population increase
Tree cutting/deforestation
Bushfires
#GNAFY10
Human population increase
Tree cutting/deforestation
Bushfires
#GNAFO11
Human population increase
Soil fertility decline
Tree cutting/deforestation
Bushfires
#GNAMY12
Bushfires
Chemical fertilizer use
Tree cutting/deforestation
#GNAFY13
Tree cutting/deforestation
Scarcity of farming lands

#GNAMI14
Scarcity of farming lands
Bushfires
Continuous farming/weeding
Tree cutting/deforestation
#GNAMO15
Tree cutting/deforestation
Continuous farming/weeding
Bushfires
Scarcity of farming lands
#GNAMO16
Scarcity of farming lands
Rainfall variability
Reproduction of grasses and trees and crops
#GNAFI17
Reproduction of grasses and trees and crops
Bushfires
Continuous farming/weeding
Tree cutting/deforestation
#GNAFY18
Tree cutting/deforestation
Bushfires
Continuous farming/weeding
#GNAFI19
Tree cutting/deforestation
Bushfires
Reproduction of grasses and trees and crops
Agricultural expansion
#GNAFY20
Agricultural expansion
Rainfall variability
Planting or protection of economic or very useful trees
Tree cutting/deforestation
Bushfires
#GNAMY21
Bushfires
Planting or protection of economic or very useful trees
Agricultural expansion
#GNAMO22
Agricultural expansion
Tree cutting/deforestation
Bushfires
Soil fertility decline
#GNAMO23
Soil fertility decline
Rainfall variability
#GNAFY24
Soil fertility decline

Bushfires
Tree cutting/deforestation
#GNAMY25
Tree cutting/deforestation
Bushfires
#GNAFO26
Bushfires
Tree cutting/deforestation
Continuous farming/weeding
#GNAFO27
Bushfires
Tree cutting/deforestation
Soil fertility decline
#GNAFO28
Soil fertility decline
Scarcity of farming lands
Tree cutting/deforestation
Bushfires
#GNAFI29
Bushfires
Rainfall variability
Tree cutting/deforestation
#GNAFO30
Tree cutting/deforestation
Soil fertility decline
Bushfires
#MNOFY01
Bushfires
Agricultural expansion
Tree cutting/deforestation
Human population increase
Chemical fertilizer use
#MNOFY02
Agricultural expansion
Human population increase
Chemical fertilizer use
Rainfall variability
#MNOMO03
Rainfall variability
Use of chemicals to spray grasses
Agricultural expansion
Human population increase
Tree cutting/deforestation
#MNOMI04
Agricultural expansion
Human population increase
Rainfall variability
Tree cutting/deforestation

Destruction of trees by elephants
#MNOMO05
Rainfall variability
Human population increase
Tree cutting/deforestation
#MNOMI06
Human population increase
Rainfall variability
Establishment of Forest reserve
#MNOMO07
Human population increase
Rainfall variability
Overgrazing
Bushfires
Destruction of trees by elephants
#MNOMI08
Human population increase
Establishment of Forest reserve
#MNOMO09
Human population increase
Agricultural expansion
Animal population increase
#MNOMY10
Human population increase
Agricultural expansion
Animal population increase
#MNOMY11
Human population increase
Agricultural expansion
Animal population increase
Bushfires
Tree cutting/deforestation
#MNOFY12
Agricultural expansion
Human population increase
Planting or protection of economic or very useful trees
#MNOFI13
Human population increase
Planting or protection of economic or very useful trees
Agricultural expansion
Use of chemicals to spray grasses
#MNOFI14
Use of chemicals to spray grasses
Good soils
Human population increase
Planting or protection of economic or very useful trees
#MNOMY15
Planting or protection of economic or very useful trees

Soil fertility decline
#MNOFY16
Planting or protection of economic or very useful trees
Bushfires
Rainfall variability
#MNOFY17
Bushfires
Rainfall variability
Natural phenomenon
#MNOFO18
Rainfall variability
#MNOFO19
Rainfall variability
Bushfires
Overgrazing
Tree cutting/deforestation
Agricultural expansion
#MNOFO20
Agricultural expansion
Bushfires
Overgrazing
#MNOFO21
Bushfires
Overgrazing
Agricultural expansion
#MNOFI22
Agricultural expansion
Rainfall variability
Tree cutting/deforestation
#MNOFI23
Rainfall variability
Bushfires
Use of chemicals to spray grasses
Agricultural expansion
#MNOFI24
Agricultural expansion
Rainfall variability
#MNOFO25
Rainfall variability
Tree cutting/deforestation
Bushfires
#MNOMI26
Rainfall variability
#MNOMI27
Rainfall variability
Agricultural expansion
#MNOMY28
Bushfires

Drought
#MNOMO29
Bushfires
Drought
Agricultural expansion
#MNOMY30
Bushfires
Agricultural expansion
High temperatures (too much sun)
Drought
Tree cutting/deforestation
Overgrazing
Rainfall variability
#MJGFO01
Bushfires
Tree cutting/deforestation
Drought
#MJGFO02
Tree cutting/deforestation
Bushfires
Drought
Infrastructural development(houses)
Agricultural expansion
#MJGFO03
Bushfires
Tree cutting/deforestation
Drought
Infrastructural development(houses)
#MJGFI04
Tree cutting/deforestation
Bushfires
Drought
#MJGFI05
Tree cutting/deforestation
Drought
Bushfires
#MJGFI06
Tree cutting/deforestation
Bushfires
Drought
#MJGFY07
Drought
Bushfires
Tree cutting/deforestation
#MJGFY08
Infrastructural development(houses)
Tree cutting/deforestation
Drought

Bushfires
#MJGMI09
Tree cutting/deforestation
Agricultural expansion
Bushfires
Drought
#MJGMY10
Drought
Tree cutting/deforestation
Agricultural expansion
#MJGMI11
Tree cutting/deforestation
Drought
Human population increase
#MJGMI12
Drought
Tree cutting/deforestation
#MJGMO13
Drought
#MJGMO14
Tree cutting/deforestation
Drought
#MJGMO15
Drought
Tree cutting/deforestation
#MJGMY16
Drought
Tree cutting/deforestation
#MJGMY17
Tree cutting/deforestation
Drought
#MJGFY18
Tree cutting/deforestation
Drought
Rainfall variability
Soil fertility decline
#MJGFI19
Drought
Agricultural expansion
Soil fertility decline
Tree cutting/deforestation
#MJGFY20
Tree cutting/deforestation
Good soils
#MJGFI21
Reproduction of grasses and trees and crops
No interest in growing some particular plants
Agricultural expansion

#MJGFO22
Agricultural expansion
Drought
#MJGFO23
Drought
#MJGMI24
Agricultural expansion
Drought
Tree cutting/deforestation
No interest in growing some particular plants
#MJGMI25
Agricultural expansion
Drought
Overgrazing
Planting or protection of economic or very useful trees
Tree cutting/deforestation
#MJGMY26
Overgrazing
Agricultural expansion
Endemic species
Tree cutting/deforestation
#MJGMY27
Tree cutting/deforestation
Agricultural expansion
Bushfires
Natural phenomenon
#MJGMO28
Bushfires
Infrastructural development(houses)
Rainfall variability
Agricultural expansion
Tree cutting/deforestation
#MJGFY29
Infrastructural development(houses)
Drought
#MJGMO30
Drought
Human population increase
Tree cutting/deforestation
#MRAFI01
Tree cutting/deforestation
Human population increase
Drought
Overgrazing
Tree cutting/deforestation
#MRAMY02
Drought
Overgrazing

Tree cutting/deforestation
#MRAFY03
Tree cutting/deforestation
Drought
#MRAFY04
Drought
Tree cutting/deforestation
#MRAMY05
Drought
Tree cutting/deforestation
#MRAFY06
Drought
Agricultural expansion
#MRAFI07
Agricultural expansion
Tree cutting/deforestation
Drought
#MRAMI08
Tree cutting/deforestation
Bushfires
Drought
Agricultural expansion
Infrastructural development(houses)
#MRAMI09
Drought
Tree cutting/deforestation
#MRAMY10
Drought
Agricultural expansion
#MRAMO11
Tree cutting/deforestation
Drought
Infrastructural development(houses)
#MRAFO12
Tree cutting/deforestation
Overgrazing
Drought
Bushfires
#MRAMI13
Tree cutting/deforestation
Drought
#MRAFO14
Tree cutting/deforestation
Drought
#MRAFO15
Tree cutting/deforestation
Drought
Human population increase

#MRAFO16
Tree cutting/deforestation
Human population increase
Drought
Bushfires
#MRAFO17
Drought
Human population increase
Tree cutting/deforestation
#MRAFY18
Drought
Tree cutting/deforestation
#MRAFI19
Drought
Tree cutting/deforestation
Agricultural expansion
Infrastructural development(houses)
#MRAFI20
Drought
Tree cutting/deforestation
#MRAFI21
Drought
Tree cutting/deforestation
Bushfires
#MRAMO22
Bushfires
Overgrazing
Tree cutting/deforestation
Planting or protection of economic or very useful trees
Agricultural expansion
Human population increase
#MRAMO23
Agricultural expansion
Human population increase
Tree cutting/deforestation
Overgrazing
Agricultural expansion
Drought
#MRAMO24
Infrastructural development(houses)
Drought
#MRAMY25
Drought
Tree cutting/deforestation
Use of chemicals to spray grasses
#MRAFY26
Drought
Tree cutting/deforestation

Agricultural expansion
#MRAMI27
Tree cutting/deforestation
Drought
Agricultural expansion
Good soils
Reproduction of grasses and trees and crops
Rainfall variability
Endemic species
#MRAMI28
Planting or protection of economic or very useful trees
Overgrazing
Drought
Tree cutting/deforestation
Agricultural expansion
#MRAMY29
Agricultural expansion
Rainfall variability
Drought
Tree cutting/deforestation
No interest in growing some particular plants
#MRAMO30
Rainfall variability
Drought
Overgrazing
Agricultural expansion
#MSIFY01
Agricultural expansion
Drought
Tree cutting/deforestation
#MSIMI02
Drought
Tree cutting/deforestation
#MSIMY03
Drought
Tree cutting/deforestation
#MSIFI04
Tree cutting/deforestation
Drought
#MSIMI05
Drought
Tree cutting/deforestation
#MSIMY06
Drought
Rainfall variability
Tree cutting/deforestation
#MSIFO07
Drought

Tree cutting/deforestation
#MSIFY08
Drought
#MSIFY09
Drought
Tree cutting/deforestation
#MSIMY10
Tree cutting/deforestation
Agricultural expansion
Drought
Reproduction of grasses and trees and crops
#MSIFO11
Drought
Agricultural expansion
#MSIMO12
Drought
Tree cutting/deforestation
#MSIMO13
Tree cutting/deforestation
Drought
#MSIFO14
Drought
Overgrazing
Tree cutting/deforestation
#MSIFO15
Drought
Tree cutting/deforestation
#MSIFO16
Drought
Tree cutting/deforestation
#MSIFY17
Drought
Tree cutting/deforestation
#MSIFI18
Drought
Tree cutting/deforestation
#MSIFI19
Tree cutting/deforestation
Drought
#MSIMI20
Drought
Tree cutting/deforestation
#MSIMO21
Tree cutting/deforestation
Drought
Agricultural expansion
Non-development of science
#MSIMI22

Drought
#MSIFY23
Drought
#MSIFY24
Drought
Agricultural expansion
#MSIMI25
Drought
Agricultural expansion
Tree cutting/deforestation
#MSIMI26
Drought
Overgrazing
#MSIFI27
Drought
Soil fertility decline
#MSIMO28
Drought
Soil fertility decline
Poverty
#MSIMO29
Drought
Poverty
Agricultural expansion
Rainfall variability
Tree cutting/deforestation
Overgrazing
#MSIMY30
Tree cutting/deforestation
Drought
Overgrazing
Agricultural expansion
Rainfall variability
#MBOFI01
Drought
Tree cutting/deforestation
#MBOFI02
Drought
Tree cutting/deforestation
#MBOFY03
Drought
Tree cutting/deforestation
#MBOFY04
Drought
Tree cutting/deforestation
Rainfall variability
#MBOFY05
Drought

Soil fertility decline
#MBOFY06
Drought
Soil fertility decline
Overgrazing
Agricultural expansion
#MBOFY07
Drought
Rainfall variability
Tree cutting/deforestation
#MBOMO08
Rainfall variability
Tree cutting/deforestation
Overgrazing
Agricultural expansion
Drought
#MBOMO09
Drought
Overgrazing
Tree cutting/deforestation
#MBOMO10
Drought
#MBOMI11
Drought
Tree cutting/deforestation
#MBOMY12
Drought
#MBOMY13
Drought
Agricultural expansion
Tree cutting/deforestation
#MBOMY14
Drought
#MBOMI15
Drought
#MBOFI16
Drought
Tree cutting/deforestation
#MBOMY17
Drought
#MBOMI18
Drought
Bushfires
Tree cutting/deforestation
#MBOMO19
Tree cutting/deforestation
Drought
Bushfires

#MBOMI20
Drought
Tree cutting/deforestation
Bushfires
#MBOMO21
Drought
Tree cutting/deforestation
#MBOMI22
Drought
Tree cutting/deforestation
Bushfires
#MBOMY23
Drought
Bushfires
#MBOFI24
Drought
Bushfires
Tree cutting/deforestation
#MBOFO25
Drought
Tree cutting/deforestation
#MBOFO26
Drought
Bushfires
Tree cutting/deforestation
#MBOFI27
Drought
Tree cutting/deforestation
#MBOFO28
Tree cutting/deforestation
Drought
Bushfires
#MBOFO29
Drought
Bushfires
Tree cutting/deforestation
#MBOFO30
Tree cutting/deforestation
Drought
Bushfires
#MSNMY01
Drought
Agricultural expansion
#MSNMY02
Drought
Overgrazing
Agricultural expansion
#MSNMI03

Overgrazing
Drought
Tree cutting/deforestation
Agricultural expansion
Rainfall variability
#MSNMI04
Drought
Agricultural expansion
Overgrazing
#MSNMO05
Overgrazing
Drought
Tree cutting/deforestation
Agricultural expansion
Rainfall variability
#MSNMO06
Drought
Tree cutting/deforestation
#MSNMO07
Drought
Agricultural expansion
Tree cutting/deforestation
Rainfall variability
#MSNMO08
Rainfall variability
Agricultural expansion
Overgrazing
Drought
Tree cutting/deforestation
#MSNMY09
Overgrazing
Tree cutting/deforestation
Drought
#MSNMY10
Tree cutting/deforestation
Drought
Bushfires
#MSNMY11
Tree cutting/deforestation
Drought
Bushfires
#MSNMO12
Drought
Bushfires
Tree cutting/deforestation
#MSNMI13
Drought
#MSNMI14

Drought
Tree cutting/deforestation
#MSNMI15
Drought
Tree cutting/deforestation
#MSNFY16
Tree cutting/deforestation
Drought
Overgrazing
Agricultural expansion
#MSNFY17
Drought
Tree cutting/deforestation
Natural phenomenon
Infrastructural development(houses)
Destruction of plants by wind
#MSNFO18
Destruction of plants by wind
Drought
Tree cutting/deforestation
#MSNFI19
Tree cutting/deforestation
Drought
#MSNFI20
Drought
Tree cutting/deforestation
#MSNFI21
Tree cutting/deforestation
Drought
#MSNFY22
Tree cutting/deforestation
Drought
Bushfires
#MSNFY23
Tree cutting/deforestation
Drought
#MSNFY24
Drought
Tree cutting/deforestation
Bushfires
#MSNFI25
Drought
Overgrazing
Tree cutting/deforestation
Agricultural expansion
#MSNFI26
Drought
Agricultural expansion

#MSNFO27
Drought
#MSNFO28
Drought
Tree cutting/deforestation
Overgrazing
#MSNFO29
Drought
Overgrazing
#MSNFO30
Drought
Agricultural expansion

ii) Free list of way forward

#DSAMO01

Crop rotational farming

#DSAMO02

Stop bushfires

Stop cutting of trees

Government intervention

#DSAMO03

Stop bushfires

Government intervention

#DSAMO04

Stop bushfires

#DSAMO05

Maintenance of farmlands

Stop cutting of trees

#DSAFI06

Stop cutting of trees

#DSAFI07

Growing more crops

#DSAFO08

Afforestation

#DSAFY09

Irrigation

#DSAMI10

No solution for natural forage but for 'artificial' forage

Community assistance

#DSAMY11

Government intervention

#DSAMI12

No solution for natural forage but for 'artificial' forage

Community assistance

#DSAMY13

God's intervention

Irrigation

#DSAMI14

Stop bushfires

Government intervention

#DSAMY15

Government intervention

#DSAMI16

Proper storage of crop residues

#DSAMY17

Proper storage of crop residues

#DSAMY18

Afforestation

#DSAMI19

Stop bushfires

Stop cutting of trees

Stop/moderate use of weedicides

#DSAFI20

Government intervention

#DSAFO21

More rainfall

#DSAFI22

More rainfall

Protection of grasses and trees for livestock

#DSAFO23

Stop bushfires

#DSAFY24

More rainfall

#DSAFY25

Afforestation

#DSAFI26

Stop bushfires

Afforestation

#DSAFO27

Stop bushfires

Government intervention

Stop cutting of trees

#DSAFY28

Prunning the trees

#DSAFY29

Prunning the trees

#DSAFO30

More rainfall

#DCHMY01

Maintenance of farmlands

Animals should be allowed to feed on grasses

#DCHMO02

God's intervention

#DCHMI03

Stop bushfires

#DCHMI04

Stop bushfires

#DCHMY05

No idea

#DCHMO06

No idea

#DCHMI07

Stop bushfires

Stop cutting of trees

Migration of strangers

#DCHMO08

Afforestation

Government intervention

#DCHMY09

No clear regulation on use of lands for grazing

#DCHMY10

Balance between grazingland and cropland proportions

Stop bushfires

Establishment of watchdog committees to fight bushfires

#DCHMY11

No idea

#DCHFO12

Application of chemical fertilizer

Fallow management/shifting cultivation

#DCHMI13

Afforestation

Stop cutting of trees

#DCHFI14

Stop cutting of trees

#DCHFY15

No idea

#DCHMO16

Application of organic manure

Application of chemical fertilizer

#DCHMO17

Application of chemical fertilizer

#DCHMI18

Crop rotational farming

#DCHFO19

More rainfall

Prunning the trees

#DCHFO20

Government intervention

Stop cutting of trees

#DCHFO21

Stop cutting of trees

#DCHFI22

Government intervention

Stop cutting of trees

#DCHFI23

Government intervention

#DCHFO24

Stop cutting of trees

#DCHFI25

Government intervention

Stop cutting of trees

#DCHFY26

Government intervention

Stop cutting of trees

#DCHFY27

Growing more crops

#DCHFY28

Government intervention

#DCHFI29

Proper storage of crop residues

#DCHFY30

Stop bushfires

Government intervention

#DJEMO01

Stop bushfires

#DJEMY02

Stop bushfires

Stop cutting of trees

Stop/moderate use of weedicides

#DJEMY03

Maintenance of farmlands

#DJEMI04

God's intervention

Afforestation

Stop bushfires

Government intervention

#DJEMY05

Stop bushfires

#DJEMY06

Government intervention

#DJEMO07

Stop bushfires

#DJEMO08

Chiefs intervention

#DJEMO09

Stop bushfires

#DJEMO10

Stop cutting of trees

Government intervention

#DJEFY11

Afforestation

#DJEFY12

No idea

#DJEFI13

Stop cutting of trees

#DJEFI14

More rainfall

#DJEFO15

Stop/moderate use of weedicides

#DJEFY16

Growing more crops

#DJEFY17

Stop bushfires

More rainfall

#DJEFI18

No idea

#DJEMY19

Government intervention

More rainfall

Application of chemical fertilizer

#DJEMY20

Afforestation

#DJEMY21

Proper storage of crop residues

#DJEFY22

Afforestation

#DJEFO23

Stop bushfires

Stop cutting of trees

#DJEFY24

Afforestation

Fallow management/shifting cultivation

Stop compound farming

Growing more crops

#DJEMI25

Stop bushfires

Stop cutting of trees

Afforestation

#DJEMI26

No idea

#DJEFO27

Fallow management/shifting cultivation

Stop bushfires

#DJEFI28

Proper storage of crop residues

#DJEFO29

More rainfall

#DJEFO30

More rainfall

Application of chemical fertilizer

#DNBMY01

Growing more crops

#DNBMI02

Afforestation

#DNBMI03

God's intervention

#DNBMI04

Government intervention

Stop cutting of trees

#DNBMI05

Public education

#DNBMO06

God's intervention

#DNBMO07

Growing more crops

#DNBMY08

Stop cutting of trees

Stop bushfires

#DNBMO09

Government intervention

Stop cutting of trees

#DNBMO10

Praying for more money

Stop bushfires

#DNBMO11

Stop bushfires

Stop cutting of trees

Growing more crops

#DNBMI12

Stop bushfires

Stop cutting of trees

#DNBFY13

Fallow management/shifting cultivation

Stop cutting of trees

#DNBFO14

Crop rotational farming

#DNBFI15

Crop rotational farming

#DNBFY16

Afforestation

#DNBFI17

No idea

#DNBFO18

Application of chemical fertilizer

#DNBFI19

No idea

God's intervention

#DNBFO20

More rainfall

#DNBFI21

More rainfall

#DNBFI22

God's intervention

#DNBFY23

Afforestation

More rainfall

#DNBFY24

More rainfall

#DNBFO25

No idea

#DNBFO26

No idea

#DNBFY27

More rainfall

#DNBMY28

Stop bushfires

Stop cutting of trees

#DNBMY29

Stop bushfires

#DNBMY30

Stop/moderate use of weedicides

Stop overgrazing of some grasses

#DKPMI01

Stop/moderate use of weedicides

#DKPMY02

Stop/moderate use of weedicides

Stop cutting of trees

Stop bushfires

#DKPMY03

Stop cutting of trees

Afforestation

#DKPFY04

Afforestation

#DKPFY05

Afforestation

Application of chemical fertilizer

#DKPFO06

Government intervention

#DKPFO07

Growing more crops

#DKPFO08

God's intervention

#DKPFI09

God's intervention

#DKPFO10

Government intervention

#DKPMY11

No idea

#DKPMO12

No idea

#DKPMI13

No idea

#DKPMI14

Government intervention

#DKPFI15

No idea

#DKPMO16

More rainfall

#DKPMO17

Fallow management/shifting cultivation

#DKPMY18

No idea

#DKPMI19

No idea

#DKPMI20

No idea

#DKPMY21

Stop bushfires

#DKPMO22

Proper storage of crop residues

#DKPMO23

More rainfall

#DKPMO24

God's intervention

#DKPFY25

God's intervention

#DKPFO26

More rainfall

#DKPFI27

God's intervention

#DKPFI28

Afforestation

#DKPFY29

More rainfall

#DKPFY30

Stop overgrazing of some grasses

#GANFY01

Afforestation

#GANMO02

God's intervention

#GANMI03

Stop cutting of trees

Afforestation

Public education

#GANMO04

Afforestation

#GANMO05

Afforestation

#GANFI06

God's intervention

#GANMO07

Government intervention

#GANFY08

Balance between grazingland and cropland proportions

#GANMO09

Afforestation

#GANFY10

No idea

#GANFO11

God's intervention

Irrigation

#GANFY12

Tithering of livestock in farming season

#GANFY13

God's intervention

#GANFY14

God's intervention

#GANFI15

Tithering of livestock in farming season

People should obey customs in the village

#GANFI16

Early sowing of crops

God's intervention

#GANMY17

Tithering of livestock in farming season

People should obey customs in the village

#GANMO18

People should obey customs in the village

#GANFI19

People should obey customs in the village

#GANFY20

Application of organic manure

Maintenance of farmlands

#GANFI21

Afforestation

Growing more crops

God's intervention

#GANFO22

God's intervention

Tithering of livestock in farming season

#GANFY23

Balance between grazingland and cropland proportions

#GANFY24

Stop cutting of trees

#GANFI25

Stop cutting of trees

#GANMY26

Growing more crops

Government intervention

Stop cutting of trees

#GANMY27

Application of chemical fertilizer

Afforestation

#GANFY28

God's intervention

Irrigation

#GANMO29

God's intervention

#GANMO30

God's intervention

#GANFI31

God's intervention

#GANMI32

God's intervention

#GANMY33

Stop bushfires

Stop cutting of trees

Public education

#GANMY34

Afforestation

Public education

Establishment of watchdog committees to fight bushfires

#GANFY35

No idea

#GANFI36

Government intervention

#GANFI37

Government intervention

#GANMY38

God's intervention

#GANMY39

Maintenance of farmlands

#GANMY40

God's intervention

#GANMO41

God's intervention

#GANMI42

People should obey customs in the village

#GANMY43

God's intervention

#GANMI44

Afforestation

#GANFI45

No idea

#GANMY46

No idea

#GANFI47

God's intervention

#GANMY48

People should obey customs in the village

#GANFY49

God's intervention

#GANFO50

Maintenance of farmlands

Application of chemical fertilizer

#GANFY51

God's intervention

#GANFY52

God's intervention

#GANFY53

God's intervention

#GANMY54

God's intervention

#GANFO55

God's intervention

#GANFI56

God's intervention

Funeral performance should not be delayed

#GANMO57

God's intervention

#GANFO58

No idea

#GANFO59

God's intervention

#GANMY60

Application of organic manure

Fallow management/shifting cultivation

God's intervention

#GANMO61

Irrigation

#GANMO62

Stop bushfires

Irrigation

#GANMI63

Establishment of watchdog committees to fight bushfires

Public education

#GANFI64

Stop bushfires

#GANFI65

No idea

#GANFO66

People should obey customs in the village

#GANMI67

God's intervention

#GANMI68

Stop cutting of trees

Maintenance of farmlands

#GANMI69

God's intervention

Government intervention

#GANFI70

God's intervention

#GANMI71

Stop/moderate use of weedicides

People should obey customs in the village

#GANMO72

God's intervention

#GANMO73

Irrigation

#GANMO74

God's intervention

#GANFI75

God's intervention

Application of organic manure

Maintenance of farmlands

#GANMY76

Afforestation

Stop cutting of trees

Establishment of watchdog committees to fight bushfires

Public education

#GWKMI01

Afforestation

Growing more crops

#GWKMY02

Application of chemical fertilizer

#GWKMI03

Afforestation

#GWKMI04

Growing more crops

#GWKMI05

Government intervention

Destruction by elephants
#GWKMO06
Stop cutting of trees
Application of chemical fertilizer
Afforestation
#GWKMO07
Afforestation
Government intervention
Destruction by elephants
#GWKMO08
Afforestation
Government intervention
Destruction by elephants
#GWKFY09
Afforestation
#GWKFY10
Afforestation
Destruction by elephants
Stop bushfires
#GWKFO11
Destruction by elephants
#GWKMI12
Afforestation
#GWKFO13
Afforestation
Stop/moderate use of weedicides
#GWKFI14
Stop cutting of trees
Stop bushfires
#GWKFO15
Irrigation

#GWKFI16

Irrigation

#GWKFO17

Irrigation

#GWKFY18

Irrigation

#GWKMY19

Irrigation

#GWKMO20

Establishment of watchdog committees to fight bushfires

#GWKMY21

Afforestation

#GWKFI22

No idea

#GWKMY23

Growing more crops

#GWKFI24

Irrigation

#GWKMO25

Irrigation

#GWKFY26

Afforestation

#GWKFY27

Stop cutting of trees

Stop bushfires

#GWKFI28

Stop cutting of trees

Stop bushfires

#GWKFO29

Stop cutting of trees

Stop bushfires

#GWKMY30

Afforestation

Application of chemical fertilizer

Stop bushfires

#GKOMI01

Afforestation

Government intervention

#GKOFY02

More rainfall

Stop bushfires

Afforestation

#GKOFY03

More rainfall

Stop bushfires

Afforestation

#GKOFO04

Afforestation

Decrease the number of animals by selling some

#GKOFO05

Afforestation

#GKOFO06

Afforestation

Decrease the number of animals by selling some

#GKOFI07

Decrease the number of animals by selling some

Government intervention

#GKOFI08

Decrease the number of animals by selling some

Afforestation

#GKOFI09

More rainfall

Stop bushfires

Government intervention

#GKOFY10

Afforestation

Stop bushfires

Stop cutting of trees

#GKOFY11

Afforestation

#GKOFI12

Afforestation

#GKOFO13

Afforestation

#GKOMY14

Application of chemical fertilizer

Afforestation

#GKOMY15

Application of chemical fertilizer

Afforestation

Stop bushfires

#GKOMY16

Afforestation

Stop cutting of trees

#GKOMY17

Afforestation

Maintenance of farmlands

Stop bushfires

#GKOMI18

Afforestation

#GKOMO19

Decrease the number of animals by selling some

#GKOMO20

No idea

#GKOMO21

No idea

#GKOMO22

Stop cutting of trees

#GKOMI23

No idea

#GKOMI24

No idea

#GKOMO25

Decrease the number of animals by selling some

Stop overgrazing of some grasses

#GKOFY26

Stop cutting of trees

#GKOFO27

Stop fulanis from migrating to our village with many cows

#GKOFI28

Stop bushfires

#GKOMI29

Stop bushfires

#GKOMY30

Establishment of watchdog committees to fight bushfires

#GTIFI01

Government intervention

Stop bushfires

Establishment of watchdog committees to fight bushfires

Stop fulanis from migrating to our village with many cows

#GTIFO02

Application of chemical fertilizer

Afforestation

#GTIFI03

Application of chemical fertilizer
Decrease the number of animals by selling some
Stop bushfires
Stop cutting of trees
God's intervention
#GTIFO04
Afforestation
Decrease the number of animals by selling some
#GTIFO05
Application of chemical fertilizer
Afforestation
Stop bushfires
Decrease the number of animals by selling some
#GTIFY06
Application of chemical fertilizer
Afforestation
Stop bushfires
Decrease the number of animals by selling some
#GTIFO07
Afforestation
Application of chemical fertilizer
#GTIMY08
Afforestation
Application of chemical fertilizer
#GTIFI09
Application of chemical fertilizer
Stop bushfires
#GTIFI10
Stop bushfires
Stop cutting of trees
Government intervention

#GTIMO11

Application of chemical fertilizer

Afforestation

Stop bushfires

#GTIMY12

Stop bushfires

Afforestation

#GTIMY13

Application of organic manure

Stop bushfires

Afforestation

#GTIMI14

Afforestation

Application of chemical fertilizer

#GTIMY15

Public education

Government intervention

Afforestation

#GTIMI16

Stop bushfires

Stop cutting of trees

#GTIMI17

Stop bushfires

Stop fulanis from migrating to our village with many cows

Afforestation

Application of chemical fertilizer

#GTIMO18

Stop bushfires

Stop cutting of trees

#GTIMY19

Afforestation

Stop bushfires

#GTIMO20

Stop bushfires

Establishment of watchdog committees to fight bushfires

Maintenance of farmlands

#GTIMO21

Stop bushfires

Stop cutting of trees

#GTIMI22

Stop bushfires

Stop cutting of trees

#GTIFO23

Stop bushfires

Stop cutting of trees

#GTIFY24

Afforestation

#GTIFY25

No idea

#GTIFY26

Stop cutting of trees

#GTIFY27

Stop cutting of trees

Public education

Stop cutting of trees

#GTIMO28

Stop cutting of trees

Stop bushfires

#GTIMI29

Stop bushfires

#GTIFI30

Maintenance of farmlands

Afforestation

#GNAMO01

Early bushfires

#GNAMI02

No idea

#GNAMI03

No idea

No idea

#GNAMI05

Afforestation

#GNAMY06

Change of scattered housing setup

Balance between grazingland and cropland proportions

#GNAMY07

No idea

#GNAFI08

Afforestation

Stop bushfires

Application of organic manure

Irrigation

#GNAFI09

Afforestation

Stop bushfires

Application of organic manure

Irrigation

#GNAFY10

Stop cutting of trees

Application of chemical fertilizer

Afforestation

Early bushfires

#GNAFO11

Stop cutting of trees

Application of chemical fertilizer

Afforestation

Early bushfires

#GNAMY12

Afforestation

Stop cutting of trees

Application of chemical fertilizer

#GNAFY13

Early bushfires

Stop cutting of trees

Fallow management/shifting cultivation

#GNAMI14

Early bushfires

Stop cutting of trees

Stop bushfires

#GNAMO15

Stop bushfires

Stop cutting of trees

Early bushfires

#GNAMO16

Stop bushfires

Stop cutting of trees

Early bushfires

#GNAFI17

Afforestation

Early bushfires

Fallow management/shifting cultivation

#GNAFY18

Growing more crops

Afforestation

Fallow management/shifting cultivation

#GNAFI19

Afforestation

Stop cutting of trees

Stop bushfires

Early bushfires

#GNAFY20

Afforestation

Stop cutting of trees

Growing more crops

Application of chemical fertilizer

#GNAMY21

Stop bushfires

Stop cutting of trees

Application of chemical fertilizer

#GNAMO22

Application of chemical fertilizer

#GNAMO23

Stop cutting of trees

Application of chemical fertilizer

Early bushfires

#GNAFY24

Stop cutting of trees

Afforestation

Stop bushfires

Fallow management/shifting cultivation

#GNAMY25

Early bushfires

Stop bushfires

Afforestation

Stop cutting of trees

#GNAFO26

Stop cutting of trees

Fallow management/shifting cultivation

Early bushfires

#GNAFO27

Stop cutting of trees

Application of chemical fertilizer

Afforestation

Early bushfires

#GNAFO28

Afforestation

Stop cutting of trees

Early bushfires

#GNAFI29

Application of chemical fertilizer

Early bushfires

Stop cutting of trees

#GNAFO30

Stop cutting of trees

Early bushfires

#MNOFY01

Stop cutting of trees

#MNOFY02

No idea

#MNOMO03

Stop cutting of trees

Stop/moderate use of weedicides

#MNOMI04

Stop cutting of trees

#MNOMO05

Stop cutting of trees

#MNOMI06

Stop cutting of trees

Stop bushfires

#MNOMO07

Public education

Afforestation

#MNOMI08

Stop bushfires

Government intervention

#MNOMO09

Afforestation

#MNOMY10

Afforestation

#MNOMY11

Afforestation

#MNOFY12

Afforestation

Stop/moderate use of weedicides

#MNOFI13

Afforestation

Application of chemical fertilizer

#MNOFI14

Maintenance of farmlands

Application of organic manure

#MNOMY15

Afforestation

Public education

#MNOFY16

Application of chemical fertilizer

Afforestation

#MNOFY17

More rainfall

#MNOFO18

Stop bushfires

Public education

Stop cutting of trees

#MNOFO19

Afforestation

#MNOFO20

Afforestation

#MNOFO21

Maintenance of farmlands

Application of chemical fertilizer

#MNOFI22

Public education

#MNOFI23

Afforestation

#MNOFI24

Afforestation

#MNOFO25

Afforestation

#MNOMI26

Public education

Afforestation

#MNOMI27

Afforestation

Public education

#MNOMY28

Public education

Proper storage of crop residues

#MNOMO29

Afforestation

Public education

#MNOMY30

Afforestation

Public education

#MJGFO01

Stop bushfires

Stop cutting of trees

#MJGFO02

Stop bushfires

Stop cutting of trees

#MJGFO03

Stop bushfires

Stop cutting of trees

#MJGFI04

Stop bushfires

Stop cutting of trees

#MJGFI05

Stop bushfires

Stop cutting of trees

#MJGFI06

Stop bushfires

Stop cutting of trees

#MJGFY07

Stop bushfires

Stop cutting of trees

#MJGFY08

Stop bushfires

Stop cutting of trees

#MJGMI09

Stop bushfires

Stop cutting of trees

#MJGMY10

Stop bushfires

Stop cutting of trees

#MJGMI11

Stop bushfires

Stop cutting of trees

#MJGMI12

Stop bushfires

Stop cutting of trees

#MJGMO13

Stop bushfires

Stop cutting of trees

#MJGMO14

Stop bushfires

Stop cutting of trees

#MJGMO15

Stop bushfires

Stop cutting of trees

#MJGMY16

Stop bushfires

Stop cutting of trees

#MJGMY17

Stop bushfires

Stop cutting of trees

#MJGFY18

Stop bushfires

Stop cutting of trees

#MJGFI19

Stop bushfires

Stop cutting of trees

#MJGFY20

Stop bushfires

Stop cutting of trees

#MJGFI21

Stop bushfires

Stop cutting of trees

#MJGFO22

Stop bushfires

Stop cutting of trees

#MJGFO23

Stop bushfires

Stop cutting of trees

#MJGMI24

Stop bushfires

Stop cutting of trees

#MJGMI25

Stop bushfires

Stop cutting of trees

#MJGMY26

Stop bushfires

Stop cutting of trees

#MJGMY27

Stop bushfires

Stop cutting of trees

#MJGMO28

Stop bushfires

Stop cutting of trees

#MJGFY29

Stop bushfires

Stop cutting of trees

#MJGMO30

Stop bushfires

Stop cutting of trees

#MRAFI01

Stop bushfires

Stop cutting of trees

#MRAMY02

Stop bushfires

Stop cutting of trees

#MRAFY03

Stop bushfires

Stop cutting of trees

#MRAFY04

Stop bushfires

Stop cutting of trees

#MRAMY05

Stop bushfires

Stop cutting of trees

#MRAFY06

Stop bushfires

Stop cutting of trees

#MRAFI07

Stop bushfires

Stop cutting of trees

#MRAMI08

Stop bushfires

Stop cutting of trees

#MRAMI09

Stop bushfires

Stop cutting of trees

#MRAMY10

Stop bushfires

Stop cutting of trees

#MRAMO11

Stop bushfires

Stop cutting of trees

#MRAFO12

Stop bushfires

Stop cutting of trees

#MRAMI13

Stop bushfires

Stop cutting of trees

#MRAFO14

Stop bushfires

Stop cutting of trees

#MRAFO15

Stop bushfires

Stop cutting of trees

#MRAFO16

Stop bushfires

Stop cutting of trees

#MRAFO17

Stop bushfires

Stop cutting of trees

#MRAFY18

Stop bushfires

Stop cutting of trees

#MRAFI19

Stop bushfires

Stop cutting of trees

#MRAFI20

Stop bushfires

Stop cutting of trees

#MRAFI21

Stop bushfires

Stop cutting of trees

#MRAMO22

Stop bushfires

Stop cutting of trees

#MRAMO23

Stop bushfires

Stop cutting of trees

#MRAMO24

Stop bushfires

Stop cutting of trees

#MRAMY25

Stop bushfires

Stop cutting of trees

#MRAFY26

Stop bushfires

Stop cutting of trees

#MRAMI27

Growing more crops

Stop bushfires

Stop cutting of trees

#MRAMI28

Stop/moderate use of weedicides

Stop bushfires

Stop cutting of trees

#MRAMY29

Stop/moderate use of weedicides

Stop bushfires

Stop cutting of trees

#MRAMO30

Stop/moderate use of weedicides

Stop bushfires

Stop cutting of trees

#MSIFY01

Stop bushfires

Stop cutting of trees

#MSIMI02

Stop bushfires

Stop cutting of trees

#MSIMY03

Stop bushfires

Stop cutting of trees

#MSIFI04

Stop bushfires

Stop cutting of trees

#MSIMI05

Stop bushfires

Stop cutting of trees

#MSIMY06

Stop bushfires

Stop cutting of trees

#MSIFO07

Stop bushfires

Stop cutting of trees

#MSIFY08

Stop bushfires

Stop cutting of trees

#MSIFY09

Stop bushfires

Stop cutting of trees

#MSIMY10

Stop bushfires

Stop cutting of trees

#MSIFO11

Stop bushfires

Stop cutting of trees

#MSIMO12

Stop bushfires

Stop cutting of trees

#MSIMO13

Stop bushfires

Stop cutting of trees

#MSIFO14

Stop bushfires

Stop cutting of trees

#MSIFO15

Stop bushfires

Stop cutting of trees

#MSIFO16

Stop bushfires

Stop cutting of trees

#MSIFY17

Stop bushfires

Stop cutting of trees

#MSIFI18

Stop bushfires

Stop cutting of trees

#MSIFI19

Stop bushfires

Stop cutting of trees

#MSIMI20

Stop bushfires

Stop cutting of trees

#MSIMO21

Stop bushfires

Stop cutting of trees

#MSIMI22

Stop bushfires

Stop cutting of trees

#MSIFY23

Stop bushfires

Stop cutting of trees

#MSIFY24

Stop bushfires

Stop cutting of trees

#MSIMI25

Stop bushfires

Stop cutting of trees

#MSIMI26

Stop bushfires

Stop cutting of trees

#MSIFI27

Stop bushfires

Stop cutting of trees

#MSIMO28

Stop bushfires

Stop cutting of trees

#MSIMO29

Stop bushfires

Stop cutting of trees

#MSIMY30

Stop bushfires

Stop cutting of trees

#MBOFI01

Stop bushfires

Stop cutting of trees

#MBOFI02

Government intervention

#MBOFY03

Government intervention

#MBOFY04

No idea

#MBOFY05

No idea

#MBOFY06

Government intervention

Balance between grazingland and cropland proportions

#MBOFY07

Irrigation

#MBOMO08

Government intervention

#MBOMO09

Government intervention

#MBOMO10

Maintenance of farmlands

#MBOMI11

Maintenance of farmlands

#MBOMY12

Maintenance of farmlands

#MBOMY13

Maintenance of farmlands

#MBOMY14

Maintenance of farmlands

#MBOMI15

Maintenance of farmlands

#MBOFI16

Maintenance of farmlands

#MBOMY17

Maintenance of farmlands

#MBOMI18

Maintenance of farmlands

#MBOMO19

Maintenance of farmlands

#MBOMI20

Maintenance of farmlands

#MBOMO21

Maintenance of farmlands

#MBOMI22

Maintenance of farmlands

#MBOMY23

Maintenance of farmlands

#MBOFI24

Maintenance of farmlands

#MBOFO25

Maintenance of farmlands

#MBOFO26

Maintenance of farmlands

#MBOFI27

Maintenance of farmlands

#MBOFO28

Maintenance of farmlands

#MBOFO29

Maintenance of farmlands

#MBOFO30

#MSNMY01

Government intervention

#MSNMY02

Government intervention

#MSNMI03

Government intervention

#MSNMI04

More rainfall

#MSNMO05

More rainfall

#MSNMO06

More rainfall

#MSNMO07

No idea

#MSNMO08

More rainfall

#MSNMY09

More rainfall

#MSNMY10

More rainfall

#MSNMY11

More rainfall

#MSNMO12

More rainfall

#MSNMI13

More rainfall

#MSNMI14

More rainfall

#MSNMI15

More rainfall

#MSNFY16

Government intervention

#MSNFY17

Government intervention

#MSNFO18

Government intervention

#MSNFI19

Government intervention

#MSNFI20

Government intervention

#MSNFI21

Government intervention

#MSNFY22

Government intervention

#MSNFY23

Government intervention

#MSNFY24

Government intervention

#MSNFI25

Government intervention

#MSNFI26

Government intervention

#MSNFO27

Government intervention

#MSNFO28

Government intervention

#MSNFO29

Government intervention

#MSNFO30

Government intervention
